# AN ADAPTIVE RADIATION OF FROGS IN A SOUTHEAST ASIAN ISLAND ARCHIPELAGO

**DOI:** 10.1111/evo.12145

**Published:** 2013-06-17

**Authors:** David C Blackburn, Cameron D Siler, Arvin C Diesmos, Jimmy A McGuire, David C Cannatella, Rafe M Brown

**Affiliations:** 1 Department of Ecology and Evolutionary Biology and Biodiversity Institute, University of KansasLawrence, Kansas, 66045; 2 Current address: Department of Vertebrate Zoology and Anthropology, California Academy of SciencesSan Francisco, California, 94118; 4 Section of Integrative Biology, University of Texas and Texas Natural Science CenterAustin, Texas, 78712; 5 Current address: Department of Biology, University of OklahomaNorman, Oklahoma, 73072; 6 Herpetology Section, Zoology Division, National Museum of the Philippines, Padre Burgos Avenue, Ermita 1000Manila, Philippines; 7 Department of Integrative Biology, University of CaliforniaBerkeley, California, 94720

**Keywords:** Comparative methods, disparity, diversification, ecomorphology, *Kaloula*, microhylidae

## Abstract

Living amphibians exhibit a diversity of ecologies, life histories, and species-rich lineages that offers opportunities for studies of adaptive radiation. We characterize a diverse clade of frogs (*Kaloula*, Microhylidae) in the Philippine island archipelago as an example of an adaptive radiation into three primary habitat specialists or ecotypes. We use a novel phylogenetic estimate for this clade to evaluate the tempo of lineage accumulation and morphological diversification. Because species-level phylogenetic estimates for Philippine *Kaloula* are lacking, we employ dense population sampling to determine the appropriate evolutionary lineages for diversification analyses. We explicitly take phylogenetic uncertainty into account when calculating diversification and disparification statistics and fitting models of diversification. Following dispersal to the Philippines from Southeast Asia, *Kaloula* radiated rapidly into several well-supported clades. Morphological variation within *Kaloula* is partly explained by ecotype and accumulated at high levels during this radiation, including within ecotypes. We pinpoint an axis of morphospace related directly to climbing and digging behaviors and find patterns of phenotypic evolution suggestive of ecological opportunity with partitioning into distinct habitat specialists. We conclude by discussing the components of phenotypic diversity that are likely important in amphibian adaptive radiations.

Comparative studies of radiations reveal the commonalities of evolutionary processes that shaped the Tree of Life. By broadly framing evolutionary radiations as the accumulation of both lineages and phenotypes (Losos and Mahler 2010), comparisons among clades become focused not on the absolute number of lineages or specific types of phenotypes but rather on the tempo and mode of speciation and phenotypic evolution; these themes have long been at the heart of studies of adaptive radiations (Simpson 1953; Givnish 1997; Schluter 2000). Studies of vertebrate radiations reveal broad patterns that include the relative importance of different modes of phenotypic diversification, the roles of character displacement and ecological opportunity, and the frequency of hybridization among lineages (Schluter and McPhail 1992; Grant and Grant 1994, 2006; Schluter 2000; Streelman and Danley 2003; Butler and King 2004; Gavrilets and Losos 2009; Glor 2010; Harmon et al. 2010).

Among the diversity of evolutionary radiations, studies of adaptive radiations focus specifically on lineage accumulation and phenotypic breadth. Adaptive radiations are frequently conceptualized as the rapid accumulation of lineages through speciation accompanied by adaptation to diverse ecological niches (Schluter 2000; Gavrilets and Losos 2009; but see Glor 2010), which is typically manifest through a diversity of corresponding phenotypes (e.g., morphologies, life histories, behaviors). During the past three decades, studies of adaptive radiation have come to differentiate the diversity of lineages in a clade (sometimes called “taxonomic diversity”) from breadth of phenotypes, or disparity (for review, see Foote 1997; Wagner 2010). By melding advances in time-calibrated molecular phylogenetics with concepts developed within paleobiology, a synthetic view of adaptive radiations emerges that encompasses both extant and extinct taxa. This synthesis has a broad impact by facilitating discovery of unrecognized adaptive radiations and developing a conceptual framework applicable across evolutionary time scales.

Despite long-standing interest in vertebrate adaptive radiations, most clades of living amphibians continue to receive little attention. Whereas many studies have focused on adaptive radiation in mammals (e.g., Simpson 1953; Springer et al. 1997; Alroy 1999; van Valkenburgh 1999; Madsen et al. 2001; Slater et al. 2010), ample opportunity remains for fundamental studies of amphibian radiations. Consider, for example, that extant mammalian species diversity is surpassed by that of living amphibians, including the species-rich clade comprising neobatrachian frogs (Köhler et al. 2005; Reeder et al. 2007; AmphibiaWeb 2012). The lack of attention to adaptive radiations in living amphibians is surprising. Amphibians exhibit a tight correlation between phenotypes and habitat types (Duellman and Trueb 1986; Stebbins and Cohen 1995; McDiarmid and Altig 1999; Wells 2007; Hillman et al. 2009), and have substantial opportunity for population divergence due to limited dispersal abilities and high philopatry (Vences and Wake 2007). Taken together, these suggest that adaptive radiations may be frequently encountered among clades of living amphibians. Recent summaries (i.e., Glor 2010; Losos and Mahler 2010) focus on a single case of nonadaptive radiation in the salamander genus *Plethodon* (Kozak et al. 2006), but in fact another salamander clade is a celebrated example of adaptive radiation. Tropical plethodontid salamanders (tribe Bolitoglossini sensu Vieites et al. 2011) represent a radiation into diverse phenotypes related to habitat utilization (Wake 1966, 1987, 2009; Wake and Lynch 1976). The adaptive radiation of this clade may have been facilitated by key innovations in both life history (direct development; e.g., Wake 1966; Chippindale et al. 2004) and trophic morphology (Lombard and Wake 1986); for a recent analysis of the relationship between speciation and phenotypic diversity in plethodontids, see Rabosky and Adams (2012). Admittedly, most claims of adaptive radiation in extant amphibians are largely ad hoc, such as for Asian toads (Bufonidae; van Bocxlaer et al. 2009), Caribbean frogs (*Eleutherodactylus*; Hedges 1989a,1989b), Andean frogs (*Telmatobius*; Cei 1986), and various lineages of ranoid frogs (Bossuyt and Milinkovitch 2000; van der Meijden et al. 2005; Bossuyt et al. 2006; see also Savage 1973). Few studies of amphibian adaptive radiations have explicitly characterized the relationship between habitat utilization and morphological variation, or examined the accumulation of phenotypic diversity through time (but see Kozak et al. 2005; Setiadi et al. 2011). Unlike other vertebrates in which feeding morphologies may play a prominent role in diversification (Liem 1973; van Valkenburgh 1999; Lovette et al. 2002; van Valkenburgh et al. 2004; Westneat et al. 2005; Collar et al. 2008; but see Lombard and Wake 1986), amphibian biologists have historically emphasized life history and reproductive modes as integral to adaptive radiation (Orton 1953, 1957; Wake 1966; Duellman and Trueb 1986; Callery et al. 2001).

Here we characterize a clade of frogs in the Philippine island archipelago as an adaptive radiation. We provide the most complete inference of phylogenetic relationships of frogs in the genus *Kaloula* (family Microhylidae), assess the relationships between habitat specialization and morphological variation, and characterize the accumulation of lineages and phenotypes. *Kaloula* exhibits habitat specialists that we refer to as ecotypes, including terrestrial ground frogs, scansorial shrub frogs, and arboreal tree-hole specialists (Inger 1954; Diesmos et al. 2002). Phylogenetic inference reveals that a single clade of *Kaloula* diversified rapidly following colonization of the Philippines. We demonstrate that morphological variation within *Kaloula* is partly explained by ecotype and that during its diversification the endemic Philippine clade exhibited high and sustained levels of disparification (sensu Evans et al. 2009).

## Materials and Methods

### TAXA, GENES, AND PHYLOGENETIC ANALYSES

The DNA dataset contains extensive population-level sampling of *Kaloula* (140 individuals) from throughout Southeast Asia, and especially the Philippines, for the mitochondrial 12S and 16S ribosomal RNA genes and intervening transfer RNA for valine (∼2400 bp). We estimated phylogenetic relationships using maximum likelihood and Bayesian approaches, and estimated relative divergence times using relaxed clock methods implemented in BEAST version 1.6.2 (Drummond et al. 2011). Our approach capitalizes on the resources for genetic and phenotypic analyses obtained through biodiversity surveys and archived in museum collections. Further details are provided in the Supporting Information.

Analyses of lineage diversification and morphological evolution were conducted on a pruned version of the maximum clade credibility tree (MCCT) and the posterior distribution of trees from BEAST analyses, using a single exemplar per species, candidate species, or subspecies (hereafter referred to simply as “species”). Tests of diversification make assumptions about the speciation and extinction of species, not gene lineages. Including multiple individuals per species could lead to erroneously favoring models in which diversification increased toward the present (Pybus and Harvey 2000). Thus, we were careful to not include multiple individuals per putative species. A phylogeny based on a large sequence dataset, which is subsequently pruned, provides a more robust estimate of topology and branch lengths than does a separate analysis based on a limited number of exemplars for each species.

### TEMPORAL PATTERNS OF LINEAGE DIVERSIFICATION

We used two approaches to evaluate whether diversification rates were homogenous through time and specifically whether diversification was rapid following colonization of the Philippines. Both incorporated phylogenetic uncertainty by summarizing diversification across the posterior distribution of trees from BEAST analyses.

First, to test whether temporal patterns of lineage-accumulation remained constant through time, we fit multiple models of diversification using the function Misfits (Burbrink et al. 2012) in R version 2.15 for Mac OS X (R Development Core Team 2008). These models are ranked by Akaike information criterion (AIC) weights for each tree and summarized across the set of post–burn-in trees. We evaluated the fit of the nine coalescent-based models of Morlon et al. (2010), each of which is a unique combination of properties characterizing the probabilities of constant or changing diversity, the presence of extinction, and constant or variable rates of speciation and extinction. For Misfits analyses, we used starting parameter values of α = 0.00001 (the exponential variation in speciation rate) and λ = 0.05 (the speciation rate), assuming complete sampling of Philippine *Kaloula*.

In addition, we estimated the γ-statistic for the constant rates test (Pybus and Harvey 2000) using the APE package for R (Paradis et al. 2004). Because extinction may mislead interpretations of the γ-statistic (Rabosky and Lovette 2008; Morlon et al. 2010), our use of the γ-statistic is informed by results of fitting multiple diversification models that incorporate extinction. Significant negative values of γ suggest that internal nodes of the phylogeny are closer to the root than expected under a Yule pure-birth model; in this respect, the Yule model (no extinction) is conservative as it would tend to push nodes closer to the root than a model with extinction. If diversification occurred rapidly following colonization of the Philippines (i.e., little extinction), then γ will be significantly negative (γ < −1.645; Pybus and Harvey 2000). Although estimations of γ are predicted to differ between gene trees and species trees, gene trees likely still provide accurate estimates of γ as long as the population parameter θ is not too large (Burbrink and Pyron 2011). Because estimates of γ should ideally incorporate uncertainty in both topology and branch lengths, we calculated γ based on both the pruned MCCT as well as each of the post–burn-in trees from the BEAST analysis. In addition, because the internode distances are affected by the choice of exemplar tips, we evaluated whether γ for the pruned MCCT was significantly different from a tree in which the same number of tips had been pruned at random. Significance for this was assessed by comparing γ to a null distribution obtained by calculating γ on 1000 replicate trees in which the MCCT is randomly pruned to the same number of tips for our exemplar tree of the endemic Philippines clade (pruned 126 tips). Because sampling within endemic Philippine *Kaloula* is assumed to be near complete, we assume that incomplete taxon sampling does not affect our estimation of the γ-statistic (Pybus and Harvey 2000). Calculations of γ and associated statistical tests were performed using the gammaStat function of the APE package.

### ECOTYPES AND MORPHOLOGICAL VARIATION

To provide a framework for interpreting temporal patterns of ecomorphological diversification within *Kaloula*, we evaluated the relationship between ecotype and morphological variation. We characterized each ecotype qualitatively as a summary of information on general ecology and natural history (e.g., obligate forest species, species common in open or disturbed habitats), as well as microhabitat preference (e.g., ground species, species that perch when active, species exclusively found in tree holes; R. M. Brown, C. D. Siler, and A. C. Diesmos, pers. obs.). We also collected morphometric data to describe the shape of each species. Then, we evaluated the relationship between ecotype class and patterns of morphological variation. We classified species of *Kaloula* into three primary ecotype classes: (1) arboreal tree-hole frogs; (2) scansorial shrub frogs; and (3) ground frogs (see Table1).

**Table 1:** Summary of qualitative categorization general geographic range, ecological type, microhabitat preference, reproduction and activity patterns, morphological specialization for terrestrial/arboreal habits (degree of finer and toe tip expansion), and overall ecotype defined here (see text for details)

Taxon	Range	General ecology and microhabitat	Reproductive characteristics and activity patterns	Finger/toe tip shape	Ecotype
*K. taprobanica*	Sri Lanka	Terrestrial; forest floor, semifossorial; ephemeral pools	Males call in water following heavy rains	Wide	Ground
*K. verrucosa*	China	Terrestrial; forest floor, semifossorial; ephemeral pools	Males call in water following heavy rains	Narrow	Ground
*K. pulchra*	Sundaland	Terrestrial; open, disturbed habitat, semifossorial; ephemeral pools	Males call in water following heavy rains	Narrow	Ground
*K. mediolineata*	Thailand	Terrestrial; forest floor, semifossorial	Males call in water following heavy rains	Narrow	Ground
*K*. sp. nov.	Vietnam	Semiarboreal; forest floor and tree trunks	Males call in water following heavy rains	Wide	Ground-tree
*K*. sp. nov.	Peninsular Malaysia	Semiarboreal; forest floor and tree trunks	Males call in water following heavy rains	Wide	Ground-tree
*K. baleata*	Java	Terrestrial; open, disturbed habitat, semifossorial	Males call in water following heavy rains	Wide	Ground-tree
*K*. sp. nov.	Palawan	Arboreal; tree hole and branches	Males call from tree holes	Wide	Tree hole
*K*. sp. nov.	Sulawesi	Terrestrial; open, disturbed habitat, semifossorial	Males call in water following heavy rains	Wide	Ground
*K. walteri*	S. Luzon	Terrestrial; montane forest floor and dry stream beds, semifossorial	Males call from gravel and rock crevices in dry season	Narrow	Ground
*K. rigida*	N. Luzon	Terrestrial; forest floor semifossorial; ephemeral pools	Males call in water following heavy rains	Narrow	Ground
*K*. sp. nov.	Sibuyan	Scansorial; ephemeral pools	Males call in water following heavy rains	Wide	Shrub
*K. c. negrosensis*	Negros	Scansorial; ephemeral pools	Males call from low elevated perches above water following heavy rains	Wide	Shrub
*K. c. meridionalis*	Mindanao	Scansorial; ephemeral pools	Males call from low elevated perches or in water following heavy rains	Wide	Shrub
*K. c. conjuncta*	S. Luzon	Scansorial, ephemeral pools	Males call in water following heavy rains	Wide	Shrub
*K*. sp. nov.	Mindoro, Semirara	Scansorial; ephemeral pools	Males call from elevated perches above water	Wide	Shrub
*K. picta*	Philippines	Terrestrial; open, disturbed habitat, semifossorial, ephemeral pools	Males call in water, year round.	Narrow	Ground
*K*. sp. nov.	Samar-Leyte	Arboreal; tree holes	Males call from tree holes	Wide	Tree hole
*K*. sp. nov.	Panay	Arboreal; tree holes	Males call from tree holes	Wide	Tree hole
*K. kokacii*	Luzon	Arboreal, tree holes	Males call from tree holes	Wide	Tree hole
*K. kalingensis*	N.W. Luzon	Arboreal; tree holes	Males call from tree holes	Wide	Tree hole
*K*. sp. nov.	E. Luzon	Arboreal; tree holes	Males call from tree holes	Wide	Tree hole

We used a multivariate analysis of continuous variables to characterize morphological variation. For each species, we aimed to collect measurement data for 10 specimens; data collection was limited to males because they are more common in museum collections. Because our analysis revealed ample cryptic diversity within Philippine *Kaloula*, we limited our data collection to populations for which we are confident of the species identity. We also collected data for two *Kaloula* species (*Kaloula borealis* and *Kaloula rugifera*) not included in our phylogeny to more fully characterize the relationship between ecotype and morphological variation within the genus. However, we were unable to include in this analysis two new species (from Panay and Sulawesi, respectively), because voucher specimens could not be accessed. Sampling for each species ranged from 3 to 10 specimens (median: 8). If most morphological variation is partitioned interspecifically, this sampling will likely result in a low type I error rate (Harmon and Losos 2005).

We measured 15 continuous characters that are relevant to ecomorphology (all measured by D.C.B. to reduce interobserver bias; Lee 1990; Hayek et al. 2001; for further details, see Supporting Information). Frog limb morphology is directly related to performance relevant to ecology (e.g., Emerson 1991). Limb proportions of the fore and hind limbs are intimately related to locomotor mode with walking and burrowing species having shorter limbs and digits than those that jump and climb (Emerson 1988). Similarly, the anatomy of the hands and feet plays an important role in different locomotor modes: foot webbing is important to propulsion in water (Stahmhuis and Nauwelaerts 2005); enlarged metatarsal tubercles on the feet facilitate digging (Emerson 1971, 1976); and expanded digit tips on the hands and feet support larger toe pads that increase surface adhesion for climbing (Emerson and Diehl 1980). Finally, head shape can explain some patterns of dietary type in frogs (Emerson 1985). For each taxon, we calculated the mean value for each measurement to maximize interspecific variation relative to intraspecific variation (Harmon and Losos 2005).

We evaluated the relationship between ecotype and measurement data using a multivariate analysis of variance (MANOVA). We asked whether ecotype classes could be discriminated based on observed morphological variation for the twenty species considered. To focus this analysis on differences in shape, we followed standard procedures (e.g., Garland et al. 1992; Collar and Wainwright 2006; Revell 2009; Mahler et al. 2010) to “size-correct” fourteen measurements by calculating their residuals based on a generalized least squares linear regression (see Hansen and Bartoszek 2012) of each variable against snout–vent length (SVL; measure of body size) using the NLME package for R. We conducted a principal components analysis (PCA) based on SVL and the 14 residuals. Principal components analysis was conducted using the prcomp function of the STATS package for R and eigenvalues explaining ≥ 10% of the cumulative variance saved for analysis. We did not incorporate phylogenetic information into this analysis because we aimed to test the relationship observed in the field between ecotype and form that spurred this study, and not the influence of phylogeny per se. We then conducted a MANOVA with ecotype class as the factor (using the manova function of the STATS package in R); significance was assessed using the Wilks’ statistic. We also conducted a separate analysis using a simulation-based phylogenetic ANOVA (Garland et al. 1993) as implemented in the phy.anova function in the GEIGER package in R (Harmon et al. 2008); 1000 simulations were used to calculate the phylogenetic *P*-value. These analyses used the same ecotype classes and the ln-transformed measurement data (excluding snout-vent length) as above, as well as the pruned species-level MCCT from our BEAST analysis; we excluded the two mainland species not included in our phylogenetic analyses.

### TEMPORAL PATTERNS OF MORPHOLOGICAL DIVERSIFICATION

Using phylogenetic comparative methods, we evaluated temporal patterns of morphological change across the evolutionary history of *Kaloula*. Measurements of morphological features related to ecotype are confounded by both body size and shared phylogenetic history. These confounding effects should be accounted for before evaluating temporal patterns of morphological change across a phylogeny. Using the pruned species-level MCCT from our BEAST analysis (representing 18 of the 20 species measured earlier), we performed a phylogenetic principal component analysis on phylogenetically size-corrected shape variables using the method of Revell (2009). By accommodating the nonindependence of observations due to shared evolutionary history, this approach reduces variance of regression coefficients so as to be closer to expected type I error rates (Felsenstein 1985; Rohlf 2006). Principal component axes with eigenvalues comprising ≥ 10% of the cumulative variance were used to represent a morphospace. Species values for these phylogeny-corrected principal components scores (PC_phylo_) provided the data for analyses of morphological evolution using comparative phylogenetic methods.

Temporal patterns of morphological diversification were evaluated using the disparity-through-time (DTT) approach (Harmon et al. 2003). We calculated total disparity as the average Euclidean distance between all points in a morphospace (Ciampaglio et al. 2001), whereas relative disparities for subclades (Foote 1993) were calculated as the disparity in the subclade divided by the total disparity. High relative disparity of a subclade indicates a relatively large contribution to total observed disparity. Using an estimate of phylogeny, the DTT approach calculates mean relative disparity for all subclades in which ancestral lineages are present at times defined by each node in the ultrametric phylogenies (Harmon et al. 2003). Thus, similar to a lineage-through-time (LTT) plot, mean relative disparity for each node is used to generate a DTT plot. For these DTT analyses, we used the PC_phylo_ scores for each species and the MCCT from BEAST. To evaluate whether the observed DTT differs from expectations under a null hypothesis of Brownian motion character change, we conducted 10,000 simulations of character change; 95% confidence intervals (CI) were constructed for these simulated data (Slater et al. 2010). Analyses were conducted using the GEIGER package. The DTT curve for the observed data was evaluated qualitatively by comparison to the 95% CI for the simulated data as well as plots of average subclade disparity by node for each of the BEAST trees. We quantitatively evaluated the DTT curve by calculating the morphological disparity index (MDI; Harmon et al. 2003). The MDI is the area contained between curves of the observed relative disparity and the median relative disparity from simulations. Values of MDI greater than 0 indicate that most morphological disparity is partitioned within subclades, whereas values less than 0 indicate that most morphological disparity is partitioned between subclades. Although DTT curves and the MDI statistic allow for inferences of phenotypic evolution that circumvent issues related to inferring ancestral states (Harmon et al. 2003), these methods may still be sensitive to uncertainties in topology and branching times. Thus, we calculated MDI for each tree in the posterior samples from BEAST analyses as above for the MCCT (though using only 1,000 simulations for calculating MDI for each tree); for each tree, we calculated MDI using scores for the first three PC_phylo_ axes based on the variance–covariance matrix for that tree. We then obtained a distribution for MDI and calculated a 95% credible interval. Finally, we evaluated whether patterns of evolution differed along each PC_phylo_ by performing all of the above DTT analyses on each PC_phylo_ separately. We then tested the fit of two models of character evolution to each PC_phylo_: a random walk (modeled as Brownian motion [BM]) and a random walk with a selective optimum (modeled as Ornstein–Uhlenbeck process [OU]; Hansen 1997; Butler and King 2004). This combination of approaches identified whether changes in specific axes of morphological variation were associated with bursts of lineage diversification.

## Results

### PHYLOGENETIC RELATIONSHIPS

**Figure 1:**
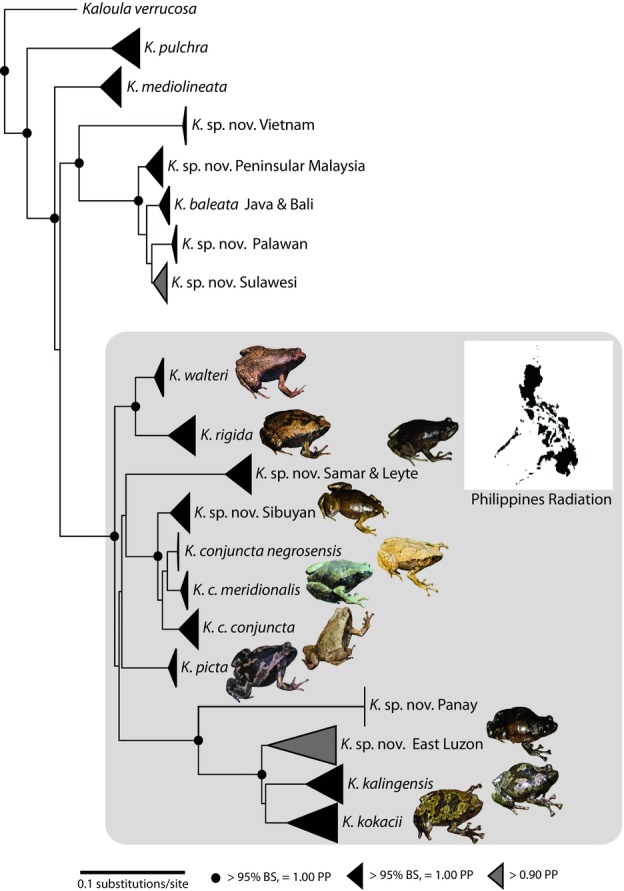
Maximum-likelihood phylogram estimated from mitochondrial DNA sequences (12S and 16S ribosomal RNA genes) depicting the phylogenetic relationships of *Kaloula* (Anura: Microhylidae; see also Fig. S1). The gray box indicates the endemic Philippine radiation. Images of each species in the Philippine radiation are provided, except for the new species from Panay.

In general, the ingroup relationships among Southeast Asian *Kaloula* are well resolved (Figs. 1, S1, S2). Both Bayesian and likelihood methods revealed strong support (PP = 1.00; ML NBS = 98%) for a large clade consisting of 12 divergent lineages endemic to the Philippines (Figs. 1, S2; Table2). Divergence time analyses suggest that an MRCA for this Philippines clade occurred in the Late Miocene (median: 11.9 mya; 95% HPD: 6.1–18.4 mya; see Supporting Information). Individuals from within this endemic Philippines radiation are assigned to five well-resolved clades: (1) *Kaloula picta*; (2) an undescribed species from Samar and Leyte islands; (3) *Kaloula rigida* + *Kaloula walteri*; (4) the “*Kaloula kalingensis*” clade (consisting of *K. kalingensis*, *Kaloula kokacii*, and two undescribed species from Panay Island and eastern Luzon Island, respectively); and (5) the “*Kaloula conjuncta*” clade (consisting of *K. conjuncta conjuncta*, *Kaloula c. meridionalis*, *Kaloula c. negrosensis*, *Kaloula c. stickeli*, and two undescribed taxa, one from Mindoro Island and another from Panay and Sibuyan islands). However, both Bayesian and ML analyses provide equivocal support (< 0.50 PP; < 50% ML NBS) for specific relationships between these five clades.

**Table 2:** Bayesian posterior probabilities (PP), maximum likelihood nonparametric bootstrap support, and average within-clade *p*-distances for recovered clades of *Kaloula*

Taxon	PP	ML NBS	*p*-distance
*K. pulchra*	1.00	100%	0.5%
*K. mediolineata*	1.00	100%	1.1%
*K*. sp. nov. Vietnam	1.00	100%	0.2%
*K*. sp. nov. Palawan	1.00	100%	0.3%
*K. baleata* Bali and Java	1.00	100%	0.8%
*K*. sp. nov. Peninsula and Borneo	1.00	100%	0.8%
*K*. sp. nov. Sulawesi	0.42	37%	1.1%
*K*. sp. nov. Samar and Leyte	1.00	100%	1.3%
*K. walteri*	1.00	100%	0.3%
*K. rigida*^1^	1.00	100%	0.5%
*K*. sp. nov. Panay	1.00	100%	0.0%
*K. kalingensis*	1.00	100%	2.3%
*K. kokacii*	1.00	100%	2.9%
*K*. sp. nov. East Luzon	0.97	75%	3.2%
*K. picta*	1.00	100%	0.2%
*K*. sp. nov. Sibuyan	1.00	75%	0.8%
*K. conjuncta conjuncta*	1.00	100%	0.7%
*K. conjuncta meridionalis*	1.00	99%	0.1%
*K. conjuncta negrosensis*	1.00	100%	0.0%

1 Calculations exclude ACD 1570.

### TEMPORAL PATTERNS OF LINEAGE DIVERSIFICATION

Rapid lineage accumulation followed colonization of the Philippines. The best-fit models of diversification for the Philippines radiation are those in which diversification varies through time (Table3). Of the coalescent-based models, the best-fit models are those with time-varying rates of speciation, with most trees fitting a pattern of expanding diversity, time-varying speciation, and no extinction (Model 6 of Morlon et al. 2010). The observed γ for the endemic Philippines radiation is strongly negative (γ for MCCT: −3.096; 95% credibility interval: −3.874, −2.181). This value of γ is significantly different than expected given random pruning the complete phylogeny (*P* < 0.01).

**Table 3:** Tests of coalescent-based models of diversification of Morlon et al. (2010). The properties of these models (saturated vs. expanding diversity; constant or varying rates; and extinction) are given; models 4a–d differs in ways in which speciation and extinction are modeled (for details, see Morlon et al. 2010). The two best-fit sets of models are ranked by mean weighted AIC scores. For each model, the frequency of models (as proportions) across the posterior distribution from BEAST analyses are given

	Model 1	Model 2	Model 3	Model 4a	Model 4b	Model 4c	Model 4d	Model 5	Model 6	AIC *w*	SD
Diversity	Saturated	Saturated	Expanding	Expanding	Expanding	Expanding	Expanding	Expanding	Expanding		
Rates	Constant	Varying	Constant	Varying	Varying	Varying	Varying	Constant	Varying		
Extinction	Positive	Positive	Positive	Positive	Positive	Positive	Positive	None	None		
Best-fit	0.0	16.7%	0.0	0.0	0.0	0.0	0.0	2.7%	75.2%	0.56	0.10
Next best	0.0	73.3%	0.0	0.0	0.0	0.0	0.0	2.0%	19.2%	0.32	0.32

### CORRELATION OF ECOTYPE AND MORPHOLOGICAL VARIATION

**Figure 2:**
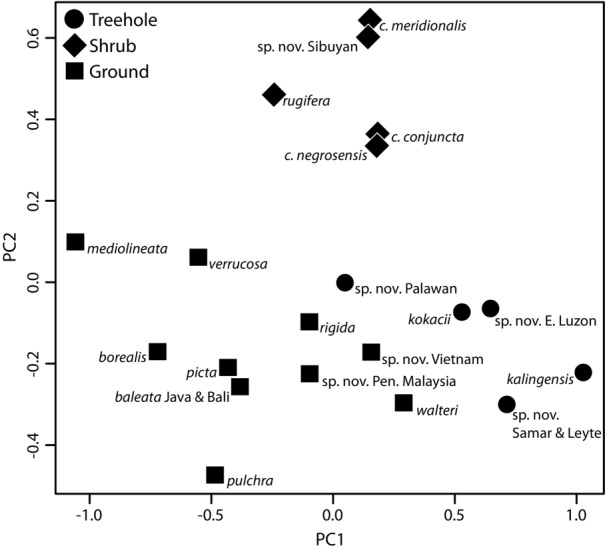
Relationship between morphological variation and ecotype categories in *Kaloula*. Species scores for the first two principal components (PC1 and PC2) are plotted; see Table4 for loadings and percent variance explained.

Principal components analysis on the size-corrected data resulted in three PC axes that each accounted for ≥ 10% of the variation. Together, the first three PC axes account for 84% of the variance (PC1: 53.1%; PC2: 20.2%; PC3: 10.6%). The strongest loadings on PC1 (Fig. 2; Table4) reveal a strong inverse correlation of the widths of finger and toe tips and the length of inner and outer metatarsal tubercles. The strongest loadings on PC2 reveal an inverse correlation between body size (SVL) and the extent of pedal webbing (with larger species having less webbing), whereas the strongest loadings on PC3 indicate a contrasting pattern with body size (SVL) being positively correlated with the extent of pedal webbing and inversely correlated with the size of the inner metatarsal tubercles. Variation in only six of 15 measurements (SVL, finger and toe tip widths, length of the inner and outer metatarsals, and extent of pedal webbing) contributes substantially to explaining patterns of interspecific morphological variation. Ecotype significantly explains patterns of variation in the morphological data. The MANOVA with ecotype as a factor is significant based on species scores on the first three PC axes (Wilks’ λ = 0.0650; *P* < 0.0001). Examination of scatter plots of PC scores also reveals general correspondence of ecotype with particular regions of morphospace (Fig. 2). The phylogenetic MANOVA is marginally nonsignificant (phylogenetic *P* = 0.0890), although this analysis included only eighteen of the twenty species that were included in the nonphylogenetic MANOVA.

**Table 4:** Loadings and variance explained by principal component analysis on size-corrected shape variables

Trait	PC1	PC2	PC3
Snout–vent length	−0.25	−0.30	−0.45
Head width	0.02	−0.07	−0.05
Snout length	0.12	0.05	0.15
Forearm length	0.06	0.13	0.08
Third finger length	0.07	0.15	−0.07
Third finger width	0.19	0.17	0.16
Third finger-tip width	0.61	0.14	−0.21
Thigh length	0.07	0.09	0.13
Crus length	0.09	0.09	0.16
Third toe length	0.08	0.16	0.15
Third toe width	0.15	0.10	0.22
Third toe-tip width	0.36	0.18	0.19
Inner metatarsal tubercle length	−0.36	0.14	0.54
Outer metatarsal tubercle length	−0.37	0.20	0.23
Webbing	0.24	−0.82	0.46
Variance explained	0.53	0.20	0.11

### TEMPORAL PATTERNS OF MORPHOLOGICAL DIVERSIFICATION

In the principal components analysis using the variance–covariance matrix from the phylogeny (MCCT), the first three principal component axes (PC_phylo_) each have eigenvalues accounting for more than 10% (12%–31%) of the cumulative variation (Table5). Together these three PC_phylo_ axes explain approximately 70% of the cumulative variation and general patterns of relative loadings are similar to analyses earlier.

**Table 5:** Loadings and variance explained by phylogenetic principal component analysis on phylogenetically size-corrected shape variables

Trait	PC1_phylo_	PC2_phylo_	PC3_phylo_
Snout–vent length	−0.17	0.58	0.31
Head width	−0.23	−0.59	−0.63
Snout length	−0.79	−0.04	−0.34
Forearm length	−0.27	0.74	−0.11
Third finger length	−0.44	0.48	0.23
Third finger width	−0.88	0.18	0.34
Third finger-tip width	−0.88	−0.26	0.17
Thigh length	−0.12	0.82	−0.41
Crus length	−0.25	0.70	−0.63
Third toe length	−0.40	0.65	−0.24
Third toe width	−0.67	−0.07	0.45
Third toe-tip width	−0.88	−0.08	0.23
Inner metatarsal tubercle length	0.55	0.57	0.15
Outer metatarsal tubercle length	0.69	0.38	0.36
Webbing	−0.15	−0.74	−0.19
Variance explained	0.32	0.28	0.13

**Figure 3:**
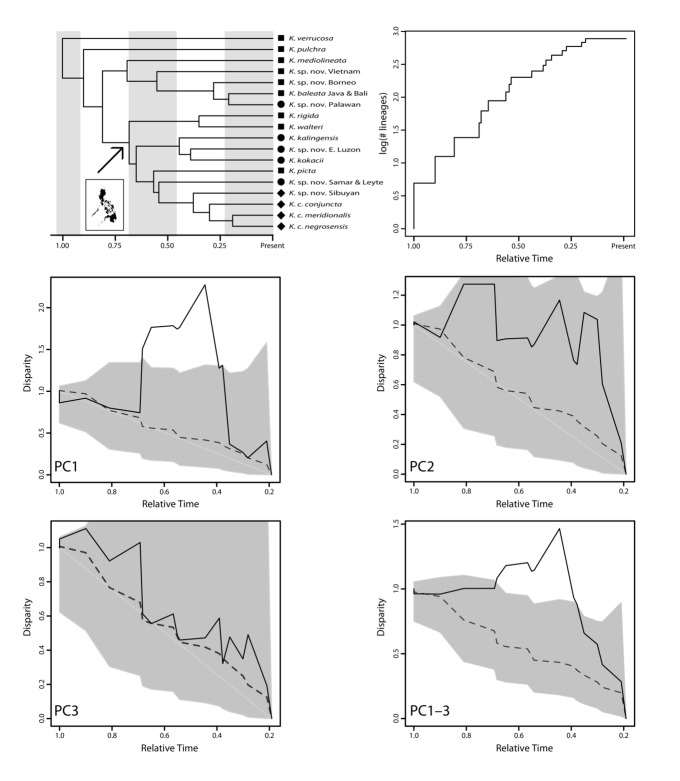
Disparification through relative time in *Kaloula*. Top-left panel shows MCCT chronogram (with shapes corresponding to ecotypes of Fig. 2) and top-right panel shows lineage-through-time plot. Middle and bottom panels show disparity-through-time (DTT) plots for PC1_phylo_, PC2_phylo_, PC3_phylo_, and all three axes together (PC1–3_phylo_). Solid black lines on DTT plots is observed disparity based on MCCT, gray lines and polygons represent median and 95% confidence intervals from BM simulations, respectively.

Disparity-through-time analyses conducted using the species scores on the PC_phylo_ axes result in different estimates of MDI depending on whether each PC_phylo_ was analyzed separately or all three together (Table6). Disparity-through-time plots for the scores on the first three axes reveal a significant increase in disparity during a period of time of active cladogenesis within the Philippines radiation (Fig. 3). During this period, average subclade disparity exceeds disparity of the clade as a whole, indicating that species within these subclades are more different from each other, on average, than species in the group as a whole. Separate analysis of scores on each PC_phylo_ axis reveals that this increase in morphological disparity is driven largely by PC1_phylo_. Visual inspection of the DTT plots for each PC_phylo_ reveals differences in the patterns of morphological evolution (Fig. 3). Fitting of BM and OU models of character evolution to these PC_phylo_ using the MCCT further supports these components as representing different evolutionary patterns (Table6). Observed disparity for PC1_phylo_ is consistent with an OU model of character change, whereas patterns are more equivocal for PC2_phylo_ and PC3_phylo_ (Table6), although the 95% CIs on the DTT plots (Fig. 3) demonstrate that observed patterns of morphological change on these two axes may be consistent with BM models.

**Table 6:** Morphological disparity index (MDI) values from disparity-through-time analyses and log-likelihoods, AICc, and Akaike weights (*w*) of Brownian motion (BM) and Ornstein-Uhlenbeck (OU) models of character evolution for each PC_phylo_ axis and all three together for MCCT from BEAST analysis

	MDI	95% CI	Range	Median	BM ln L	OU ln L	BM AICc	OU AICc	ΔAICc	BM *w*	OU *w*
PC1_phylo_	0.436	0.230–0.675	0.109–0.970	0.420	−73.686	−70.576	152.229	148.998	3.231	0.199	0.801
PC2_phylo_	0.311	0.109–0.535	−0.007–0.836	0.303	−72.465	−69.768	149.788	147.383	2.405	0.300	0.700
PC3_phylo_	0.096	0.058–0.337	−0.032–0.497	0.166	−65.328	−64.680	135.513	137.206	1.693	0.429	0.571
PC1–3_phylo_	0.312	0.200–0.457	0.118–0.586	0.323	–	–	–	–	–	–	–

## Discussion

### DIVERSIFICATION AND DISPARIFICATION

Following colonization of the Philippines island archipelago possibly in the Late Miocene (see Supporting Information), *Kaloula* rapidly diversified into several well-supported clades, each characterized by one of three distinctive ecotypes. In general, the best-fit coalescent models of diversification for the Philippines radiation are those with increasing diversity and changes in the speciation rate through time. With 12 species-level lineages, the species richness of *Kaloula* endemic to the Philippines is approximately equal to that found elsewhere in southern Asia (including the four undescribed species in the *baleata* clade). All ecotypes found within the Philippines radiation are also found in species of *Kaloula* not included in this radiation, which indicates convergent evolution. Because the Philippines radiation diversified into ecotypes found also in species of *Kaloula* from mainland Asia, this suggests constraints on ecotype diversity and/or the existence of several ecomorphological “optima.”

**Figure 4:**
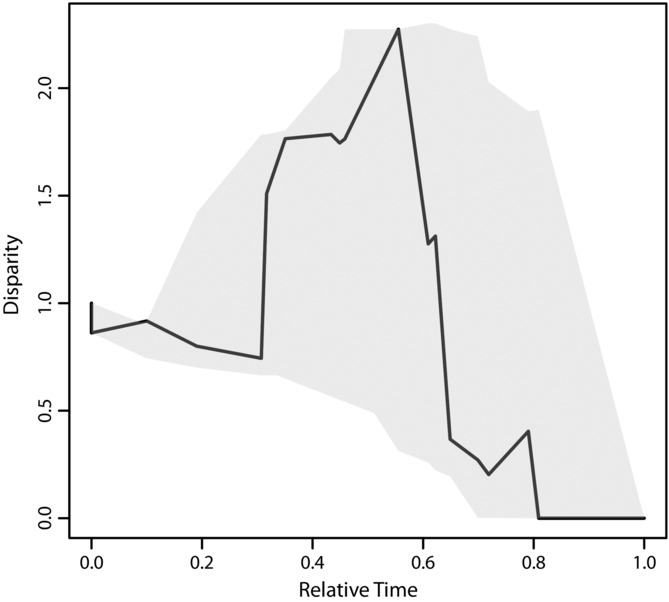
Uncertainty in disparification through time in *Kaloula* for PC1_phylo_. Solid black line on DTT plot is observed disparity based on MCCT; gray polygons represent 95% confidence interval for observed disparity based on post–burn-in trees from BEAST analysis.

During diversification, Philippine *Kaloula* exhibited high levels of disparification. The DTT analyses indicate that the disparity accumulated during the Philippines radiation lies outside of the 95% CI based on expectations using a BM model (Fig. 3). This inference appears robust to phylogenetic uncertainty (Figs. 4, S3). Furthermore, the morphological disparity index for PC1–3_phylo_ (MDI for MCCT: 0.311; 95% credibility interval: 0.118–0.586) indicates that disparity during the evolution of *Kaloula* has been on average greater than expected. During the Philippines radiation, accumulation of disparity along a single axis (PC1_phylo_) exceeds the total average subclade disparity of *Kaloula* (Fig. 3). Morphological evolution along this axis is best-fit by an OU model (a random walk with a selective optimum) rather than by a Brownian model, and represents a contrast between traits related to climbing and digging. Support for an OU model of change provides evidence of a selective optimum for this suite of traits and the loading on traits related to climbing (length and width of digits and digit tips) and digging (lengths of metatarsal tubercles) is suggestive of morphological tradeoffs involved in vertically partitioning habitats during ecotype evolution. The patterns for PC2_phylo_ and PC3_phylo_ are more equivocal.

### ECOLOGICAL OPPORTUNITY AND BIOGEOGRAPHY

The pattern observed for Philippine *Kaloula* supports the suggested role of ecological opportunity as a driving force underlying adaptive radiations (Worthington 1937, Lack 1940; Simpson 1953; Rensch 1959; see also Sepkoski 1984), particularly in island systems (Mayr 1963; Mahler et al. 2010). Although other anuran taxa may already have occupied the Philippines when *Kaloula* arrived, there are few other microhylid frogs that might have limited feeding and foraging opportunities for *Kaloula* (Inger 1954; Alcala and Brown 1998; Brown 2007). The derived feeding system of microhylid frogs is based on a muscular hydrostatic tongue that can be aimed side-to-side without moving the head (Meyers et al. 2004); this is shared only with two African frog families

Hemisotidae and  Brevicipitidae (Nishikawa  et al. 1999;  Meyers et al. 2004), and skeletal features unique to the family Microhylidae may be related to this feeding system (Trueb et al. 2011). Furthermore, microhylids tend to specialize on small prey, especially ants and termites (Emerson 1971; Das 1996; Parmalee 1999; Wells 2007). The feeding system and ecology of microhylid frogs suggest that ecological opportunities for this family might differ from that of other anurans, including in the Philippines. Aside from *Kaloula*, there are few microhylid species in the Philippines. Both *Chaperina fusca* and *Kalophrynus pleurostigma* are found on islands and the mainland of the continental Sunda Shelf; both species occur on the Mindanao Pleistocene Aggregate Island Complex (PAIC; Brown and Diesmos 2009) and *C. fusca* is also found on Palawan Island. Two species of *Oreophryne* are endemic to the Mindanao PAIC. A single species of *Microhyla* is only present on the southern island of Tawi-tawi. The endemic Philippine radiation of *Kaloula* occurs on six PAICs (Table7), and co-occurs with these other microhylids only on the Mindanao and Palawan PAICs. Furthermore, humans might have facilitated the dispersal of the only species (*K. picta*) in the Philippine radiation that occurs on Palawan Island. Thus, it seems likely that early in the endemic Philippine radiation species of *Kaloula* potentially competed with other microhylid frogs only on the Mindanao PAIC. If indeed the ecological opportunities available to microhylids do differ from those of other anurans, then there may have been substantial opportunity for diversification within *Kaloula* following colonization of the archipelago.

**Table 7:** Distribution of ecotypes by Philippine Aggregate Island Complex (PAIC)

PAIC	PAIC area (km^2^)	Ecotypes	No. Taxa	Taxa
Luzon	147,451	Ground, shrub, tree-hole	7	*K. c. conjuncta*; *K. kalingensis*; *K. kokacii*; *K. rigida*; *K. walteri*; *K. picta; K*. sp. nov. East Luzon
Mindanao	175,430	Ground, shrub, tree-hole	3	*K. c. meridionalis*; *K. picta*; *K*. sp. nov. Samar and Leyte
Mindoro	13,009	Ground, shrub	2	*K. c. conjuncta*; *K. picta*
Negros-Panay	59,623	Ground, shrub, tree-hole	3	*K. c. negrosensis*; *K. picta*; *K*. sp. nov. Panay
Palawan	61,198	Ground, tree-hole	2	*K. picta*; *K*. sp. nov. Palawan
Romblon	1,407	Ground, shrub	2	*K. picta*; *K*. sp. nov. Sibuyan

We find both a strong correlation between ecotype and morphological diversity and a partitioning of ecotypes into distinct clades during the Philippine radiation. This suggests that these three ecotypes arose early following dispersal into the archipelago with subsequent speciation predominantly mediated by allopatric isolation coupled with conservation of each clade's ancestral ecotype. Within ecotypes of the Philippine radiation, it is clear that dispersal occurred across oceanic barriers: tree-hole frogs occur on three PAICs; shrub frogs on four PAICs; and the broad distribution of the ground frog *K. picta* on many PAICs implies high levels of dispersal, and potentially human-mediated range expansion facilitated by conversion of forests to agriculture (Brown et al. 2010). The remaining ground frogs, *K. rigida* and *K. walteri*, are restricted to the Luzon PAIC. There is a rough positive relationship between PAIC size and the number of ecotypes present, with Luzon, Mindanao, and Negros-Panay PAICs possessing the greatest ecotype diversity (Table7). Yet, there is no obvious order of colonization of the islands, as observed in other systems (i.e., Hawaiian islands; Roderick and Gillespie 1998; Hormiga et al. 2003).

The relationship between phenotype and colonization ability is of interest in studies of both invasive species (Kolar and Lodge 2001) and radiations in island systems (Losos 1992; Schluter et al. 1997; Gillespie 2004; Cristescu et al. 2010), with recent work drawing parallels between these (Poe et al. 2011). Although ancestral state reconstructions for the ecotype of the MRCA of the endemic Philippine radiation are ambiguous (see Supplementary Materials), limited evidence suggests its MRCA may have been a ground frog. First, the most common ecotype outside of the endemic Philippine radiation is a ground frog (Table1). Second, it is clear that ground frogs accomplished two other dispersal events across oceanic barriers. These include the colonization of Palawan by the *Kaloula baleata* clade, which are all ground frogs except for the undescribed species endemic to Palawan, and the dispersal of the ground frog *K. picta* throughout the Philippines.

Although there is no evidence for further ecotype diversification following the initial partitioning of ecotypes by clade, morphological evolution within these subclades is above average relative to the total observed disparity in *Kaloula*. This indicates continued phenotypic diversification subsequent to the evolution of distinct ecotypes, although it remains difficult at present to evaluate the significance of within-ecotype diversification. Although ecotypes cluster in the phylogeny, species of *Kaloula* forming a community at any given locality in the Philippines are not close relatives. For example, communities at sites on the Luzon PAIC are composed of *K. picta* (large ground frog), *K. conjuncta conjuncta* (shrub frog), a *kalingensis* clade species (treehole frog), and a *rigida* clade species (small ground frog). Thus, communities of *Kaloula* species appear to exhibit phylogenetic overdispersion of structural habitat specialist phenotypes (e.g., Emerson and Gillespie 2008).

Phenotypic evolution in the Philippines radiation of *Kaloula* is potentially consistent with more than one pattern of phenotypic change. The evolution of morphological diversity along PC1_phylo_ (Fig. 3) exhibits high levels of variation within subclades and is best-fit by an OU model of change (Table6). By incorporating selection and drift, support for the OU model provides evidence for stabilizing selection and/or phenotypic constraints (e.g., Lande 1976; Felsenstein 1988; Martins 1994; Hansen 1997; Butler and King 2004; Harmon et al. 2010). Thus, our analysis may provide support that phenotypic diversification in the Philippine radiation was at least partially associated with selective optima related to ecotypes.

### ADAPTIVE RADIATIONS IN AMPHIBIANS

Given the general lack of detailed studies of adaptive radiations in amphibians, it is not surprising that few patterns have emerged specific to amphibians. Although diversification into three ecotypes may not constitute an unusual diversity of adaptive forms (e.g., Warheit et al. 1999; Losos and Miles 2002), phenotypic diversification related to habitat utilization is only one component of an adaptive radiation. We suspect that the axes of disparification in amphibians may differ from those of other vertebrate radiations. The Philippine radiation of *Kaloula* satisfies the first stage of the “general vertebrate model” of adaptive radiation, with initial diversification into habitat specialists followed by morphological diversification along other axes (Streelman and Danley 2003; see summary in Glor 2010). For many vertebrates, one important axis of subsequent disparification may be related to trophic biology, including diversity of feeding morphologies (Liem 1973; Lovette et al. 2002; van Valkenburgh et al. 2004; Westneat et al. 2005), or strategies related to behaviorally partitioning habitats (e.g., Losos 2009). Although the Philippine radiation of *Kaloula* might have had an ecological opportunity for colonization and diversification because of differences in feeding from other Philippine frogs, there is no indication of differences among these species of *Kaloula*. In fact, most adult anuran amphibians are believed to be fairly similar in trophic biology by being both predatory and feeding generalists (Nishikawa 2000). Other than the rare example of robust skulls of snail-eating frogs (Drewes and Roth 1981) and the general observation that species specializing on ants have relatively narrower heads (Toft 1980, 1981; Emerson 1985), there has been little study of the dietary relevance of morphological diversity in structures such as the tongue (Emerson 1985; Grant et al. 1997; but see Trueb and Gans 1983). Resource partitioning is documented in anurans, but this may be due more to differences in habitat and less associated with selectivity of prey types (Toft 1985).

We suggest that for amphibians, especially the order Anura (i.e., frogs and toads), partitioning of the environment in relation to reproductive modes and life histories plays an important role in adaptive radiation. This is suggested both by past discussions of radiations in amphibians that emphasize the importance of life history (Orton 1953, 1957; Duellman and Trueb 1986), and observations of clades with high levels of life history diversity (Wells 2007). Two promising lines of future inquiry on adaptive radiations in anurans are evaluating (1) the relationships between lineage accumulation and the diversity of reproductive modes and life histories (Gomez-Mestre et al. 2012); and (2) the relationship between diversification and either resource partitioning among tadpoles (Heyer 1973, 1974; Toft 1985) or by different reproductive modes (Salthe and Duellman 1973).

## Conclusions

Across frog diversity, it is difficult to characterize any clade as having an unusually great diversity of adaptive forms. Yet, interestingly, the same forms have evolved again and again throughout the > 200 million year history of crown-group Anura. It is clear that there is convergence in ecomorphological form across vast spans of time, with many last sharing a common ancestor more than 50 million years ago and some more than 200 million years ago (Roelants et al. 2007). Examples of remarkable convergence in morphology among taxa that last shared a common ancestor in the Mesozoic include aquatic frogs such as *Barbourula* (Bombinatoridae) and *Conraua* (Conrauidae), large terrestrial frogs such as *Leptobrachium* (Megophyridae) and *Astylosternus* (Arthroleptidae), small aposematic terrestrial frogs such as the diverse species in the Dendrobatidae and Mantellidae, generalized terrestrial frogs in Strabomantidae and Ceratobatrachidae, burrowing “spade-foot” frogs such as *Pelobates* (Pelobatidae) and *Heleioporus* (Limnodynastidae), and fossorial frogs such as *Rhinophrynus* (Rhinophrynidae), *Hemisus* (Hemisotidae), and *Nasikabatrachus* (Nasikabatrachidae). Because of their long evolutionary history, ecomorphological diversity, and life-history characteristics, frogs represent a remarkable research opportunity for studying how the same suites of phenotypes might have evolved at different periods during the Mesozoic and Cenozoic. Studying phenotypic evolution within discrete clades such as our study of the genus *Kaloula* or even within families (such as the Mantellidae endemic to Madagascar) will be fruitful. However, an even broader net should be cast to examine patterns of convergence and disparification across many clades as there have likely been many periods of rapid diversification and disparification in anurans in both the Mesozoic and Cenozoic. Furthermore, although broad categories of form are recognizable and possibly related to locomotion, diet, and habitat type (Duellman and Trueb 1986; Emerson 1988; Hillman et al. 2009), there is a need for field studies that explicitly relate performance and fitness to morphological and life history features characteristic of the diverse frog ecotypes. As phylogenetic resolution improves within the anuran Tree of Life, studies of frogs promise many opportunities for understanding phenotypic diversification and the partitioning of environments in relation to reproductive modes and life history.

**Associate Editor: P. Lindenfors**

## Supporting Information

**Table S1.** Summary of specimens corresponding to genetic samples included in the study. **Figure S1.** Maximum-likelihood phylogram estimated from mitochondrial DNA sequences (12S and 16S ribosomal RNA genes) depicting the phylogenetic relationships of other microhylid taxa in relation to *Kaloula.* **Figure S2.** Maximum-likelihood phylogram estimated from mitochondrial DNA sequences (12S and 16S ribosomal RNA genes) depicting the phylogenetic relationships of *Kaloula* (Anura: Microhylidae; figure complements Fig. S1). **Figure S3.** Uncertainty in disparification through time in *Kaloula*.
