# Supplementary material for: AN ADAPTIVE RADIATION OF FROGS IN A SOUTHEAST ASIAN ISLAND ARCHIPELAGO
Source: Evolution. 2013 Jun 17;67(9):2631–46. doi: 10.1111/evo.12145 (PMC3920640; doi:10.1111/evo.12145)
Supplement: Supplementary file 1 — Table S1. Summary of specimens corresponding to genetic samples included in the study. Figure S1. Maximum-likelihood phylogram estimated from mitochondrial DNA sequences (12S and 16S ribosomal RNA genes) depicting the phylogenetic relationships of other microhylid taxa in relation to Kaloula. Figure S2. Maximum-likelihood phylogram estimated from mitochondrial DNA sequences (12S and 16S ribosomal RNA genes) depicting the phylogenetic relationships of Kaloula (Anura: Microhylidae; figure complements Fig. S1). Figure S3. Uncertainty in disparification through time in Kaloula. [file evo0067-2631-sd1.pdf]

AN ADAPTIVE RADIATION OF FROGS IN A SOUTHEAST ASIAN ISLAND ARCHIPELAGO

David C. Blackburn, Cameron D. Siler, Arvin C. Diesmos, Jimmy A. McGuire, David C. Cannatella, and Rafe M. Brown

SUPPLEMENTARY TEXT

Materials and Methods

TAXON SAMPLING

Fieldwork was conducted by RMB, CDS, ACD, JAM and colleagues throughout Southeast Asia between 1992 and 2008. Tissue samples were flash-frozen in liquid nitrogen, or immersed in ≥ 90% ethanol or in a tissue buffer, then stored at -80°C. Most specimens are deposited in the Natural History Museum of the University of Kansas (KU), the National Museum of the Philippines (PNM), the Texas Memorial Museum of University of Texas at Austin (TNHC), or the Museum of Vertebrate Zoology of the University of California at Berkeley (MVZ; Supplementary Table 1). To avoid issues regarding uncertainty in interpreting results of phylogenetic inference because of including DNA sequence data from tissue samples lacking voucher specimens (Yates 1985; Winker et al. 1996; Ruedas et al. 2000; Lehn et al. 2007), we examined voucher specimens corresponding to 98% of all in-group specimens sampled and employed a strict method of species identification based on external morphology, microhabitat, and advertisement call (Diesmos et al. 2002; Brown et al. 2006a,b), which is consistent with widely accepted lineage-based species concepts (Wiley, 1978; de Queiroz, 1998, 1999).

Our DNA dataset is based on sequences from 172 specimens of frogs of the family Microhylidae, including members of 17 outgroup genera. Because our goals were to estimate the phylogeny of the Southeast Asian genus *Kaloula* and previous phylogenetic analyses have not definitively established the sister taxon to this genus (e.g., Bossuyt et al. 2006; Frost et al. 2006; van Bocxlaer et al. 2006; van der Meijden et al. 2007; Matsui et al. 2011; Trueb et al. 2011), we sampled broadly across Asian microhylid genera, including eight of nine recognized genera of the Microhylinae (sensu van der Meijden et al. 2007; no genetic samples were available for *Uperodon*), as well as members of several New World genera; the brevicipitid *Callulina* was used as an outgroup. The ingroup sampling included 140 specimens of the genus *Kaloula*, assigned to eleven of the fifteen formally recognized species (Inger 1954; Alcala and Brown 1998; Brown 2007). Genetic resources were unavailable for several currently recognized species (*K. assamensis*, *K. borealis*, and *K. rugifera*); one of these (*K. assamensis*) remains poorly known and was described on the basis of only a few specimens (Das et al. 2005). Because the taxonomic status of two additional species, *K. aureata* and *K. macrocephala*, remains unclear, we do not include them in our study (Ohler 2003; Pauwels and Chérot 2006; IUCN 2012). Our sampling is especially dense in the Philippines (106 samples) and includes all known allopatric island populations of each species, seven of which are found naturally in the Philippines (*K. pulchra* has been introduced recently; Diesmos et al. 2005, 2006; Siler et al. 2011). This sampling includes the widespread Philippine endemic *Kaloula picta* from throughout the archipelago as well as all four subspecies of the Philippine species *K. conjuncta* (*K. c. conjuncta, K. c. meridionalis, K. c. negrosensis*, and *K. c. stickeli*; Inger 1954). Additionally, we include

nearly all known allopatric populations of the widespread *Kaloula baleata*, including samples from Java (the type locality), Bali, Sulawesi, the Togian Islands, Peninsular Malaysia, Vietnam, Borneo, and Palawan Island (Philippines). Finally, we include samples of the widespread species *Kaloula pulchra* from throughout the entirety of its native range (China, Peninsular Malaysia, Laos, Thailand, Vietnam, Sumatra, and Sulawesi). However, our sampling also includes a number of undescribed species. Further details on specimens and localities can be found in the Supplementary Table 1.

DNA DATA COLLECTION

We used either Qiagen DNeasy Tissue Kit (Cat. No. 69506) or a non-commercial guanidine thiocyanate method (Esselstyn et al. 2008) to extract genomic DNA from liver (or occasionally muscle) samples. Four primer pairs (MVZ59–Val, 12L1–16Sh, 12Sm–16Sa, and 16Sc–16Sd; Moriarty and Cannatella 2004; Darst and Cannatella 2004) were used to amplify via polymerase chain reaction (PCR) an approximately 2400 bp region of the mitochondrial genome spanning most of the 12S and 16S ribosomal RNA (rRNA) genes and the intervening transfer RNA (tRNA) for Valine. We used the now standard reaction mixtures and thermal cycle profiles for both PCR and sequencing reactions, using the same primers and following the methods of Moriarty and Cannatella (2004) and Evans et al. (2003). Samples were purified using Qiagen gel preps or Exosap purification protocols (USB Corp., Cleveland, OH, USA; Esselstyn et al. 2008). Sequencing reactions were conducted with identical undiluted PCR primers, using ABI Big Dye terminator chemistry (Perkin-Elmer, Boston, MA, USA) and Sephadex clean-ups (GE Healthcare, Uppsala, Sweden). Sequencing was performed on an ABI 3130*xl* automated PRISM

sequencer (Applied Biosystems, Foster, CA, USA). All sequences are deposited in GenBank (Supplementary Table 1); a multiple alignment file is deposited in Dryad (doi:10.5061/dryad.dj342).

Because PCR products were amplified with varying success, the full length of the target sequence (approx. 2400 bp) could not be obtained for some specimens. In most cases this resulted from failure to amplify the 5' end of our target region, namely the MCZ59–Val region; the resulting sequences are approx. 1900 bp in length. Although missing data can bias or mislead phylogenetic inference, especially when concentrated in particular taxa or certain clades, taxa with missing data still add information to phylogenetic inference and missing data will not necessarily mislead phylogenetic analyses (Wiens 2006; Wiens and Moen 2008). Our goal in including specimens with incomplete data is to place particular samples with statistical support into the phylogeny to facilitate species delimitation and identification; in this way, we increased the number of specimens represented in both our molecular and morphological datasets. We included a given sample if we obtained sequence data for at least one of the four overlapping targeted gene regions; in most cases, these incomplete sequences derived from degraded tissue samples for which only 600–700 bp of the 16Sc–16Sd region could be amplified and sequenced. Taxa with incomplete gene representation were included in our analyses to aid in the confident identification of all specimens; analyses excluding taxa with missing data provided largely similar results (data not shown).

All fragments were sequenced in both directions. Sequences were assembled and manually vetted in Sequencher 4.5 (Genecodes, Ann Arbor, MI, USA), and then initially aligned using default parameter values in Clustal X 1.83.1 (Thompson et al. 1997). Non-

overlapping DNA fragments from a single specimen were aligned separately and then merged in Mesquite 2.5 (Maddison and Maddison, 2008) to form a single terminal taxon for phylogenetic analyses. Alignments were examined exhaustively by eye and manually adjusted to minimize the number of parsimony informative change across sites (Moriarty and Cannatella 2003). Because excluding ambiguously aligned regions and autapomorphic insertion-deletions ("indels") from phylogenetic analyses resulted in minimal differences in topology and bootstrap values in exploratory parsimony searches (data not shown), we chose to maintain these sites in our analysis. The resulting alignment of 2567 bp corresponds to positions 2062–4620 of the *Xenopus laevis* mitochondrial genome (GenBank NC-001573). Uncorrected pairwise distances (*p*-distances) were calculated using MEGA 4.0.1 (Tamura et al. 2007).

PHYLOGENETIC ANALYSES

We used maximum-likelihood and Bayesian approaches to estimate phylogenetic relationships. A maximum-likelihood tree was obtained using RAxML v.7.0.4 (Stamatakis 2006) using a random starting tree, the faster rapid hill-climbing algorithm of (Stamatakis et al. 2007), and a GTR + I + Γ model of evolution (with four discrete categories for Γ); the data were not partitioned into subsets. Three hundred replicate searches were conducted to avoid basing our phylogenetic inference on a local search optimum; the tree with the best –ln likelihood from these replicate searches was selected as our maximum likelihood estimate of the phylogeny. Support for the preferred ML topology was estimated using 1000 independent nonparametric bootstrap replicates (ML BS), using the same model of sequence evolution, a random starting tree, and one search

replicate per bootstrap replicate. Split-support for clades was summarized using SumTrees in DendroPy (Sukumaran and Holder 2010) and clades present in ≥ 70% of the bootstrap trees were considered well-supported (Hillis and Bull 1993).

A Bayesian estimate of phylogeny was obtained with MrBayes v.3.1.2 (Ronquist and Huelsenbeck 2003) using the GTR + I + Γ model and unpartitioned dataset. Four replicates of four MCMC chains were run for 20 million generations, sampled every 2000 generations, using a temperature of 0.2 and default priors. We examined trends and distributions of log-likelihoods and parameter values using Tracer 1.5 (Rambaut and Drummond 2009). We assessed convergence by examining correlations of split frequencies and chain variability among independent runs using Are We There Yet? (AWTY; Nylander et al. 2008). Based on examination of trends in Tracer and correlations of split frequencies, stationarity and convergence was achieved after one million generations. However, because of patterns of chain variability during the first few million generations, we took a conservative approach and discarded trees sampled during the first ten million generations as burn-in. Based on the cumulative set of post-burn-in trees from each run, all effective samples sizes (ESS) were greater than 400. The topology and posterior probabilities (PP) were then summarized based on the post-burn-in trees using SumTrees and Tracer. We considered topologies with posterior probabilities ≥ 0.95 to be well supported (Huelsenbeck et al. 2001).

We generated a time-calibrated estimate of phylogenetic relationships to test patterns of lineage diversification and morphological evolution. Analyses were conducted using the uncorrelated relaxed clock method (Drummond et al. 2006) implemented in BEAST v.1.6.2 (Drummond et al. 2010). Phylogenetic relationships were estimated using

the Yule pure-birth speciation prior for the tree shape and a GTR + I + Γ model of sequence evolution. For comparative analyses, we generated chronograms with relative divergence times by setting a normal prior of 100 (mean: 100; standard deviation: 0.001) on the most recent common ancestor (MRCA) of all tips in the tree; the distant outgroup *Callulina* was excluded from these analyses. MCMC analyses were run for 200 million generations with MCMC steps and divergence times recorded every 5000 generations; to avoid autocorrelation between MCMC steps, the sampling was further thinned to every 10,000 generations. We then assessed stationarity of parameter estimates using Tracer and thus discarded the first 5 million generations as burn-in; all ESS values were > 190. The 95% highest posterior density interval and maximum clade credibility topology (MCCT) were calculated using TreeAnnotator (Rambaut and Drummond 2011).

While the absolute timing of divergences are of interest, there are no internal fossil calibration points for the family Microhylidae. To provide a rough estimate of divergence times, we conducted another BEAST analysis using two secondary calibration priors based on van der Meijden et al. (2007): (1) MRCA of Microhylidae at 66.0 ± 11.0 million years ago (mya); and (2) MRCA of Microhylinae at 52.0 ± 9.0 mya. A normal distribution was used for each calibration point with mean and standard deviation of the prior based on the estimates provided by van der Meijden et al. (2007); we used a normal distribution for these priors because of the bidirectionality of uncertainty in these estimates (Ho and Phillips 2009).

ECOTYPES AND MORPHOLOGICAL VARIATION

Our characterization of ecotype summarizes data on ecology and natural history, including reproductive and larval biology, and microhabitat preferences. These assessments utilize a half-century of accumulated information on the life history and biology of the frogs of interest (Inger 1954, 1966; Inger and Stuebing 1989, 1997; Zhao and Adler 1993; Dutta and Manamendra-Arachichi 1996; Manthey and Grossman 1997; Fei 1999; Inger et al. 1999; Fei and Ye 2000; Diesmos et al. 2002; Schleich and Kastle 2002; Malkmus et al. 2002), as well as our collective personal experience with many of these species.

We selected measurements of morphological features likely related to habitat utilization in anurans. For instance, we expect the relative size of limbs, toe pads, and metatarsal tubercles to be directly related to behaviors of habitat specialists such as climbing or burrowing. These measurements are standard in Asian anuran taxonomy (Inger 1954, 1966; Taylor 1962; Matsui 1984, 1994; Inger and Stuebing 1989, 1997; Zhao and Adler 1993; Dutta et al. 1996; Fei and Ye 2000; Brown and Guttman 2002; Malkmus et al. 2002) with the exception of our measure of webbing (see below). The measurements used in our analyses are as follows: snout–vent length; head width (measured at the widest point of the head, near the jaw articulation); snout length (measured from the most anterior margin of the eye to the tip of the rostrum); forearm length (measured from the most proximal margin of the elbow to the proximal margin of the most proximal palmar tubercle); third finger length (measured from the proximal margin of the most proximal subarticular tubercle to the distal digit tip); third finger width (measured at the level of the most distal subarticular tubercle); third finger-tip width (maximal width of the distalmost finger); thigh length (measured from the cloaca

to the distalmost knee); crus length (measured from the most proximal knee to the most distal tibiotarsal joint); inner metatarsal tubercle length (maximum length); outer metatarsal tubercle length (maximum length); third toe length (measured from the proximal margin of the most proximal subarticular tubercle to the distal digit tip); third toe width (measured at the level of the most distal subarticular tubercle); third toe-tip width (maximal width of the distalmost toe); and extent of pedal webbing (measured from the distal tip of the third toe to the most proximal lateral margin of the toe where intradigital webbing is attached). Typically, intradigital webbing is described using the formula developed by Savage and Heyer (1967, 1997). However, this description does not lend itself to analyses using multivariate statistics. We instead opted for a novel approach by taking a measurement in which greater values indicate less webbing; thus, for example, if a principal components axis loads strongly and positively on this variable it means that species with large positive scores have less webbing than those with negative scores. For some taxa (e.g., *K. kalingensis*), the outer metatarsal tubercle is essentially absent and thus results in a measurement of nil. Because all mean scores were subsequently natural log-transformed, we added the value of 1 to all mean scores before further calculations. We were thus able to include information from all species for all measurements even though the outer metatarsal tubercle is absent in some species. All measurement data used for these analyses are deposited in Dryad (doi:10.5061/dryad.dj342).

RECONSTRUCTION OF ANCESTRAL ECOTYPES

We evaluated patterns of change in ecotype class across the phylogeny and determine the ecotype class of the MRCA of the Philippines clade. Because of uncertainties in the phylogenetic estimate, we used Bayesian methods for reconstructing ancestral ecotype states across *Kaloula* phylogeny. In diversification analyses, we used the pruned species-level time-calibrated post-burn-in trees from our BEAST analysis to reconstruct ancestral states and analyze patterns of morphological evolution. We integrated over uncertainties in topologies using Bayesian mutational mapping as implemented in SIMMAP 1.5.2 (Bollback 2006), which employs a stochastic model of character change. SIMMAP analyses treated character states as unordered and utilized an equal prior for the bias parameter, a gamma distribution for the rate parameter prior (α = 1.25, β = 0.25, k = 60), 10 sample replicates, and 10,000 draws from the prior distribution in order to have sufficient sampling of the posterior distribution for bias and rates. We evaluated the robustness of our inference to priors for the gamma distribution by conducting similar analyses in which we varied α, k, and θ; because the results of these various analyses were qualitatively similar (data not shown), we interpret our inference as robust.

Results

PHYLOGENETIC RELATIONSHIPS

Of the 2567 sites included in the phylogenetic analyses, 1469 are variable and 1145 are parsimony informative. Below, we discuss patterns of relationships based on maximum likelihood (ML) and Bayesian analyses (including the maximum clade credibility tree [MCCT] estimated from MrBayes analyses); divergence times are derived based on BEAST analyses using secondary calibration points.

We recovered strong support for a clade consisting of Southeast Asian *Kaloula*, *Kaloula taprobanica* (Sri Lanka), *Ramanella obscura* (Sri Lanka) and the two species of the Southeast Asian genus *Metaphrynella* (PP = 1.00; ML NBS = 94%). Our divergence time analyses suggest that the MRCA of this clade occurred in the Late Paleogene (median: 31.4 mya; 95% HPD: 17.0–48.0 mya). In the ML and MCCT trees, sister to this clade is a weakly supported (PP = 0.47; ML NBS = 34%) clade of south Asian genera (*Micryletta, Glyphoglossus, Caluella, Chaperina,* and *Microhyla*) that comprise the remainder of the subfamily Microhylinae. This sister relationship between these two clades supports the monophyly of the Microhylinae albeit with low support (PP = 0.61; ML NBS = 34%). In the ML and MCCT trees, sister to the clade comprising the Microhylinae, is a clade containing the Papuan genera *Oreophryne, Cophixalus,* and *Australochaperina* plus the south Asian genus *Kalophrynus* (Supplementary Fig. 1).

We do not recover *Kaloula* as monophyletic (Supplementary Figure 1). Instead, in both the ML and MCCT trees, *K. taprobanica* (from eastern India, Bangladesh, and Sri Lanka) is recovered as the sister of a clade comprising *Metaphrynella* and *Ramanella* (recovered as sister taxa; PP = 0.94; ML NBS = 62%) and all other *Kaloula*. Based on these analyses, there is low support for alternative placements of *K. taprobanica*, including a monophyletic *Kaloula* (PP = 0.04; ML NBS = 29%) and clade containing *K. taprobanica*, *Ramanella*, and *Metaphrynella* (PP = 0.01; ML NBS = 2%). In contrast, the Bayesian analysis placed the *Ramanella* + *Metaphrynella* clade sister to the Southeast Asian members of the genus *Kaloula* (PP = 0.90; ML NBS = 38%) to the exclusion of *K. taprobanica*. Excluding *K. taprobanica*, there is unequivocal support for the monophyly of other species of *Kaloula* (PP = 1.0; ML NBS = 96%), and the MRCA of these species

probably occurred in the Late Oligocene to Early Miocene (median: 22.3 mya; 95% HPD: 11.4–33.9 mya).

There is strong support (PP = 1.00; ML NBS = 96%) for a clade containing divergent and geographically circumscribed populations typically referred to *K. baleata*, but which we consider a complex of species, some of which are obviously morphologically distinct; we refer to this as the "*baleata* clade." A new morphologically distinct species within the *baleata* clade that occurs on Palawan is the only Philippine species of *Kaloula* not derived from the primary Philippine radiation and, thus, represents a second invasion of the archipelago. Relationships of the *baleata* clade remain unclear. In the ML tree, the *baleata* clade is sister to the endemic Philippines clade (PP = 0.57; ML NBS = 44%) whereas in the MCCT tree, it is sister to the mainland species *K. mediolineata* (PP = 0.16; ML NBS = 24%). The clade containing *K. mediolineata*, the *baleata* clade, and the endemic Philippine clade receives high support (PP = 1.00; ML NBS = 96%). There is also strong support for the relationship of *K. verrucosa* (China) and *K. pulchra* (a widespread Asian species) forming successively branching taxa sister to the remaining members of *Kaloula* (Fig. 1).

Most species, subspecies, and candidate species are recovered as monophyletic with high support (Table 2). The relationships of one specimen of *K. rigida* from the type locality remains unclear, but otherwise *K. ridiga* and *K. walteri* are each recovered as monophyletic with strong support. A number of well-supported clades exist within the Philippines, though relationships between these clades remain unclear. *Kaloula rigida* and *K. walteri* are resolved as sister taxa (PP = 1.00; ML NBS = 100%). There is strong support for the monophyly of an undescribed species from Samar and Leyte islands.

Kaloula kalingensis, *K. kokacii*, and two undescribed species are recovered as a strongly supported clade (PP = 1.00; ML BS = 100%), which we refer to as the "*kalingensis*" clade.

The MRCA of the *conjuncta* (median: 4.4 mya; 95% HPD: 1.9–7.6 mya) and *rigida* + *walteri* (median: 4.8 mya; 95% HPD: 1.7–8.5 mya) clades likely both occurred in the Early Pliocene, whereas the MCRA of the *kalingensis* clade may be slightly older (median: 9.1 mya; 95% HPD: 4.5–14.4 mya). Notably, because of the overlap in 95% HPD for the ages of these clades we cannot reject the hypothesis that these clades diversified contemporaneously.

INTRASPECIFIC PATTERNS OF GEOGRAPHIC DIVERSIFICATION

Our intraspecific sampling within several species allows for a characterization of divergence within these taxa (Table 2). For reference to the values reported for other species, the mean *p*-distance across *Kaloula* (excluding *K. taprobanica*) is 7.5%. Two widespread species, *K. pulchra* (throughout Southeast Asia) and *K. picta* (throughout the Philippines) exhibit nearly identical sequences across their respective broad geographical distributions (*K. pulchra*: 0.5%; *K. picta*: 0.2%). *Kaloula baleata* consists of a highly divergent and morphologically distinct Vietnam population (> 7% divergent from other species in the "*baleata*" clade) that is sister to a clade comprising moderately divergent lineages from Borneo, the Malay Peninsula, Borneo, Java + Bali, Sulawesi, and Palawan (< 3% from one another).

Divergences among species in the *K. kalingensis* clade are an order of magnitude greater than those observed for other species of *Kaloula* (Table 2). The *kalingensis* clade

consists of four strongly supported and highly divergent clades: *K. kalingensis* from northern Luzon (including samples from close to the type locality in Kalinga-Apayao Province), *K. kokacii* from the Bicol Peninsula of southern Luzon Island (adjacent to the type locality on Catañduanes Island), and then two undescribed species from Panay Island and east Luzon Island, respectively.

Within populations assigned to *K. conjuncta*, moderate levels of divergence were detected and not all described subspecies corresponded to monophyletic entities. *Kaloula conjuncta conjuncta* from numerous localities on Luzon Island was paraphyletic with respect to a population on Mindoro Island that is morphologically and acoustically distinct (Brown et al. unpubl. data). Similarly, populations diagnosable morphologically and acoustically as *K. conjuncta negrosensis* from Panay and Negros Islands did not form a clade exclusive of other subspecies; in the ML estimate, the Panay population of *K. c. negrosensis* is most closely related to a new form from Sibuyan Island that is morphologically and acoustically distinct from all other taxa (Brown et al. unpubl. data). Specimens identified as *K. c. stickeli* were genetically identical to those identified as *K. c. meridionalis*.

Moderate levels of both geographic structure and genetic divergence were also detected within allopatric populations of the species *K. rigida* and *K. walteri*.

ECOTYPE EVOLUTION

Analysis using Bayesian mutational mapping provides an ambiguous perspective on patterns of ecotype evolution. For example, posterior probabilities of each ecotype for the

MRCA of the endemic Philippines radiation are all very similar (tree hole frog: 0.33; ground frog: 0.36; scansorial shrub frog: 0.31).

Discussion

RELATIONSHIPS, DIVERGENCES, AND TAXONOMIC IMPLICATIONS

Our analysis suggests that *Kaloula taprobanica*, a species with a range extending from Sri Lanka to Bangladesh, does not form a clade with other species of *Kaloula*. While *K. taprobanica* clearly forms a clade with *Metaphrynella*, *Ramanella*, and other species of the genus *Kaloula*, the more precise relationships of *K. taprobanica* remain unclear. Similarly, in analysis of microhylid relationships that included *K. pulchra* and *K. taprobanica*, van Bocxlaer et al. (2006) did not resolve *Kaloula* as monophyletic. Similar results were obtained by Matsui et al. (2011) and Trueb et al. (2011). Because of differences in the topology between ours and other studies, we refrain from taking taxonomic action on the generic status of *K. taprobanica*. Our findings indicate a need for further study employing greater sampling of loci and taxa within South Asian microhylid frogs.

Two contrasting patterns of genetic diversity were unexpected. Two species demonstrated almost no geographic-based genetic variation, despite extensive morphological variation and wide geographical ranges. Both *K. pulchra* (throughout Southeast Asia) and *K. picta* (throughout the Philippines) exhibit nearly identical sequences across their respective broad distributions and yet both have been the subject of much discussion by taxonomists who anticipated impending taxonomic subdivision of these species (Parker 1934; Inger 1954, 1966). Populations historically referred to

Kaloula baleata (type locality: Java) include several putative new species, including a highly divergent and morphologically distinct Vietnam population (> 7% divergent from other species in the *baleata* clade) and morphologically similar populations from Peninsular Malaysia, Java + Bali, and Sulawesi (< 3% from one another). In addition, the Philippine exemplar of the *baleata* clade (*Kaloula* sp. nov. from Palawan) is a highly distinctive, small-sized, tree-hole breeding species. In accordance with their status as distinct and diagnosable allopatric evolutionary lineages (Wiley 1978; de Queiroz 1998,1999), we consider these allopatric populations of the *baleata* clade to be distinct species that require formal description.

Divergences among species in the *kalingensis* clade are much greater than those observed between other species of *Kaloula* (Table 2). The *kalingensis* clade contains two described species (*K. kalingensis* from northern Luzon, including the type locality in Kalinga Province, and *K. kokacii* from the Bicol Peninsula of southern Luzon Island, adjacent to the type locality on Catañduanes Island), and two undescribed species from Panay Island and east Luzon Island, respectively.

Kaloula conjuncta exhibits moderate levels of divergence between clades, not all of which correspond to existing taxonomy. *Kaloula c. conjuncta* from numerous localities on Luzon Island was paraphyletic with respect to a population on Mindoro Island that is morphologically and acoustically distinct (Brown et al. unpubl. data). Similarly, populations that are morphologically and acoustically diagnosable as *K. conjuncta negrosensis* from Panay and Negros Islands did not form a clade exclusive of other subspecies; in the ML estimate, the Panay population of *K. c. negrosensis* is most

closely related to a putative new species from Sibuyan Island, the latter of which is morphologically and acoustically distinct from all other taxa (Brown et al. unpubl. data).

Mindanao specimens phenotypically similar to the type series of *K. c. stickeli* (holotype FMNH 60786) are nested within *K. c. meridionalis* and are genetically identical to this morphologically distinct taxon. *Kaloula c. stickeli* is morphologically intermediate between *K. c. meridionalis* (a smaller scansorial species with widely expanded digital disks) and *K. picta* (larger terrestrial species with non-expanded digital disks) and was originally described from northern portions of the Mindanao aggregate island complex (Samar and Leyte Islands; Inger 1954). The possibility of a hybrid origin for *K. c. stickeli* seems likely. Specimens included in our analysis were collected on Mindanao Island from a large mixed chorus of individuals of *K. c. meridionalis*, *K. picta* and those similar to *K. c. stickeli*. Interspecific amplexus was observed between *K. c. meridionalis* and *K. picta* (RMB and JAM, personal observation). Taken together, the field observations and genetic similarity between specimens identified phenotypically as *K. c. stickeli* and *K. c. meridionalis* suggest that *K. c. stickeli* is a morphologically intermediate phenotype that results from natural hybridization between these *K. c. meridionalis* and *K. picta*. As a result of these findings, and for the purposes of our comparative analyses, *K. c. stickeli* was not treated here as a valid taxon. We anticipate that future taxonomic work will place *K. c. stickeli* in the synonymy of *K. c. meridionalis*. In fact, this hybridization among species in the Philippines radiation is not surprising and fits an emerging pattern highlighting hybridization as a common and important part of radiations (Grant and Grant 2002; Seehausen 2004; Wiens et al. 2006; Cristecu et al. 2010).

Finally, moderate levels of both geographic structure and genetic divergence were also detected within allopatric populations of the species *K. rigida* and *K. walteri*. Our one sample of *K. rigida* from the type locality (Baguio City, Luzon Island) is highly divergent from the remaining *K. rigida* from the forested mountains of northern Luzon and falls out sister to *K. walteri* plus the remaining *K. rigida*. Future studies, involving targeted geographical sampling and additional sampling near Baguio City will be necessary to resolve uncertainty in the status of *K. rigida*.

In summary, several discrepancies between existing taxonomy and observed distribution of phylogenetic diversity will require an eventual comprehensive taxonomic review of species diversity—an undertaking beyond the scope of this paper. We note that the recognition of the above undescribed species will result in ~ 40% increase in known diversity in *Kaloula* (see Brown and Diesmos 2002, Brown 2007, and Brown et al. 2008 for review of rapidly changing taxonomy in Philippine anurans). This increase in diversity is unsurprising given recent similar studies of taxonomic diversity and cryptic species in Southeast Asian amphibians (Brown et al. 2008; Stuart and Bain 2008; Brown and Stuart 2012).

SUPPLEMENTARY LITERATURE CITED

Brown, R. M., and A. C. Diesmos. 2001 (2002). Application of lineage-based species concepts to oceanic island frog populations: the effects of differing taxonomic philosophies on the estimation of Philippine biodiversity. The Silliman Journal 42:133–162.

Brown, R. M., A. C. Diesmos, and A. C. Alcala. 2008. Philippine amphibian biodiversity is increasing in leaps and bounds. Pp. 82–83 *in* S. N. Stuart, M. Hoffmann, J. S. Chanson, N. A. Cox, R. Berridge, P. Ramani, and B. E. Young, eds. Threatened Amphibians of the World. Lynx Ediciones, Barcelona, Spain; IUCN, Arlington.

Brown, R. M., J. Foufopoulos, and S. J. Richards. 2006. New species of *Platymantis* (Amphibia; Anura: Ranidae) from New Britain and redescription of the poorly known Platymantis nexipus. Copeia 2006:674–695.

Brown, R. M., S. J. Richards, J. Sukumaran, and J. Foufopoulos. 2006. A new morphologically cryptic species of forest frog (genus *Platymantis*) from New Britain Island, Bismarck Archipelago. Zootaxa 1334:45–68.

Brown, R. M., and B. L. Stuart. 2012. Patterns of biodiversity discovery through time: an historical analysis of defining amphibian species boundaries in the Southeast Asian mainland and island archipelagos. Pp. 348–389 *in* D. J. Gower, K. G. Johnson, J. E. Richardson, B. R. Rosen, L. Rüber, and S. T. Williams, eds. Southeast Asian Gateway Evolution, Royal Holloway University of London, Egham, England.

Darst, C.R. and D.C. Cannatella. 2004. Novel relationships among hyloid frogs inferred from 12S and 16S mitochondrial DNA sequences. Mol. Phylogenet. Evol. 31:462–475.

Das, I., S. Sengupta, M. F. Ahmed, and S. K. Dutta. 2005. A new species of *Kaloula* (Anura: Microhylidae) from north-eastern India. Hamadryad 29:101–109.

Diesmos, A. C., R. M. Brown, and G. V. A. Gee. 2005. Preliminary report on the amphibians and reptiles of Balbalasang-Balbalan National Park, Luzon Island, Philippines. Sylvatrop 13:63–80.

Diesmos, A. C., M. L. Diesmos, and R. M. Brown. 2006. Status and distribution of alien invasive frogs in the Philippines. J. Environ. Sci. Management, Philippines 9:41–53.

de Queiroz, K. 1998. The general lineage concept of species, species criteria, and the process of speciation. Pp. 57–75 *in* D. J. Howard and S. H. Berlocher, eds. Endless Forms: Species and Speciation. Oxford University Press, New York.

de Queiroz, K. 1999. The general Lineage concept of species and the defining properties of the species category. Pp. 49–89 *in* R. A. Wilson, R. A., ed. Species: New Interdisciplinary Essays. Massachusetts Institute of Technology Press, Cambridge.

Drummond, A. J., S. Y. W. Ho, M. J. Phillips, and A. Rambaut. 2006. Relaxed phylogenetics and dating with confidence. PLoS Biology 4:e88.

Dutta S. K. and K. Manamendra-Arachchi. 1996. The Amphibian Fauna of Sri Lanka. Wildlife Heritage Trust of Sri Lanka, Sri Lanka, 230 pp.

Esselstyn, J. A., H. J. D. Garcia, M. G. Saulog, and L. R. Heaney. 2008. A new species of *Desmalopex* (Pteropodidae) from the Philippines, with a phylogenetic analysis of the Pteropodini. J. Mammal. 89: 815–825.

Evans B. J., R. M. Brown, J. A. McGuire, J. Supriatna, N. Andayani, A. Diesmos, D. Iskandar, D. J. Melnick, and D. C. Cannatella. 2003. Phylogenetics of fanged frogs: testing biogeographical hypotheses at the interface of the Asian and Australian faunal zones. Syst. Biol. 52:794–819.

Fei, L., 1999. Atlas of Amphibians of China. Publishing House for Scientific and Technological Literature, Hunan.

Fei, L., and C.-Y. Ye. 2000. The Colour Handbook of the Amphibians of Sichuan. Chengdu Institute of Biology, the Chinese Academy of Sciences, Chengdu, China, 280 pp.

Frost, D. R., T. Grant, J. Faivovich, R. H. Bain, A. Haas, C. F. B. Haddad, R. O. de Sá, A. Channing, M. Wilkinson, S. C. Donnellan, C. J. Raxworthy, J. A. Campbell, B. L. Blotto, P. Moler, R. C. Drewes, R. A. Nussbaum, J. D. Lynch, D. M. Green, and W. C. Wheeler. 2006. The amphibian tree of life. Bull. Amer. Mus. Nat. Hist. 297:1–370.

Grant, P. R., and B. R. Grant. 2002. Unpredictable evolution in a 30-year study of Darwin's finches. Science 296:707–711.

Hillis D. M, and J. J. Bull. 1993. An empirical test of bootstrapping as a method for assessing confidence in phylogenetic analysis. Syst. Biol. 42:182–192.

Ho, S. Y. W., and M. J. Phillips. 2009. Accounting for calibration uncertainty in phylogenetic estimation of evolutionary divergence times. Syst. Biol. 58:367–380.

Huelsenbeck, J. P., F. Ronquist, R. Nielsen, and J. P. Bollback. 2001. Bayesian inference of phylogeny and its impact on evolutionary biology. Science 294:2310–2314.

Inger, R. F. 1966. The systematics and zoogeography of the Amphibia of Borneo. Fieldiana 52:1–402.

Inger, R. F. and R. B. Stuebing. 1989. Frogs of Sabah. Sabah Parks Trustees, Kota Kinabalu, Sabah, Malaysia, 132 pp.

Inger, R. F. and R. B. Stuebing. 1997. A Field Guide to the Frogs of Borneo. Science and Technology Unit, Sabah, Malayisa, 205 pp.

Inger, R. F., N. Orlov, and I. S. Darevsky. 1999. Frogs of Vietnam: A report on new

collections. Fieldiana (Zool.) 92:1–46.

IUCN. 2012. IUCN Red List of Threatened Species. Version 2012.1. Accessible at <www.iucnredlist.org>.

Lehn, C., I. Das, M. R. J. Forstner, and R. M. Brown. 2007. Responsible vouchering in turtle research: an introduction and recommendations. Pp. 147–156 *in* H. B. Shaffer, N. N. FitzSimmons, A. Georges, and A. Rhodin, eds. Defining Turtle Diversity: Proceedings of a Workshop on Genetics, Ethics and Taxonomy of Fresh Water Turtles and Tortoises. Chelonian Research Monographs 4.

Maddison, W. P., and D. R. Maddison. 2008. Mesquite: a modular system for evolutionary analysis, v.2.5. <http:mesquiteproject.org>.

Malkmus, R., U. Manthey, G. Vogel, P. Hoffmann, and J. Kosuch. 2002. Amphibians and Reptiles of Mount Kinabalu (North Borneo). A. R. G. Gantner Verlag Kommanditgesellschaft, Ruggell, Germany, 424 pp.

Manthey, U., and W. Grossman. 1997. Amphibien und Reptilien Südostasiens. Natur und Tier, Verlag, Germany, 512 pp.

Matsui, M. 1984. Morphometric variation analyses and revision of the Japanese toads (Genus *Bufo*, Bufonidae). Contr. Biol. Lab, Kyoto Univ. 26:209–428.

Matsui, M. 1994. A taxonomic study of the *Rana narina* complex with descriptions of three new species (Amphibia: Ranidae). Zool. J. Linn Soc. 111:385–415.

Matsui, M., A. Hamidy, D. M. Belabut, N. Ahmad, S. Panha, A. Sudin, W. Khonsue, H.-S. Oh, H.-S. Yong, J.-P. Jiang, and K. Nishikawa. 2011. Systematic relationships of Oriental tiny frogs of the family Microhylidae (Amphibia, Anura) as revealed by mtDNA genealogy. Mol. Phylogenet. Evol. 61:167–176.

Moriarty, E. C., and D. C. Cannatella. 2004. Phylogenetic relationships of the North American chorus frogs (*Pseudacris*: Hylidae). Mol. Phylogenet. Evol. 30:409–420.

Nylander, J. A. A., J. C. Wilgenbusch, D. L. Warren, and D. L. Swofford. 2008. AWTY (are we there yet?): A system for graphical exploration of MCMC convergence in Bayesian phylogenetics. Bioinformatics 24:581–583.

Ohler, A. 2003. Comments on a new book on the Amphibia of Thailand, with a tentative allocation of the figured species. Alytes 21:100–102.

Parker, H. W. 1934. A Monograph of the Frogs of the Family Microhylidae. Trustees of the British Museum, London.

Pauwels, O. S. G., and F. Chérot. 2006. Translation of the original description of *Kaloula aureata* Nutphand, 1989 (Anura: Microhylidae), with lectotype designation. Hamadryad 30:172–175.

Rambaut, A., and A. J. Drummond. 2009. Tracer v1.5, Available at http://beast.bio.ed.ac.uk/Tracer.

Rambaut, A., and A. J. Drummond. 2011. TreeAnnotator v1.6.2, Available at http://beast.bio.ed.ac.uk/TreeAnnotator.

Ronquist, F., and J. P. Huelsenbeck. 2003. MRBAYES 3: Bayesian phylogenetic inference under mixed models. Bioinformatics 19:1572–1574.

Ruedas, L. A., J. Salazar-Bravo, J. W. Dragoo, and T. L. Yates. 2000. The importance of being earnest: what, if anything, constitutes a "specimen examined?" Mol. Phylogenet. 17:129–132.

Savage, J. M., and W. R. Heyer. 1967. Variation and distribution in the tree frog genus *Phyllomedusa* in Costa Rica, Central America. Beiträge zur Neotropischen Fauna 5:111–131.

Savage, J. M., and W. R. Heyer. 1997. Digital webbing formulae for anurans: a refinement. Herpetological Review 28:131.

Schleich, H., and W. Kastle, eds. 2002. The amphibians and reptiles of Nepal. A. G. R. Gantner Verlag Kommanditgesellschaft, Ruggel, Germany, 1211 pp.

Seehausen, O. 2004. Hybridization and adaptive radiation. Trends Ecol. Evol. 19:198–207.

Siler, C. D., L. J. Welton, J. M. Siler, ,J. Brown, A. Bucol, A. C. Diesmos, and R. M. Brown. 2011. Amphibians and reptiles, Luzon Island, Aurora Province and Aurora Memorial National Park, north Philippines: new island distribution records. Check List 7:182–195.

Stamatakis, A. 2006. RAxML-VI-HPC: maximum likelihood-based phylogenetic analyses with thousands of taxa and mixed models. Bioinformatics 22:2688–2690.

Stamatakis, A., F. Blagojevic, D. Nikolopoulos, and C. Antonopoulos. 2007. Exploring new search algorithms and hardware for phylogenetics: RAxML meets the IBM cell. Journal of VLSI Signal Processing 48:271–286.

Stuart, B. L., and R. H. Bain. 2008. Amphibian species discovery in mainland Southeast Asia. Pp. 83–84 *in* S. N. Stuart, M. Hoffmann, J. S. Chanson, N. A. Cox, R. J. Berridge, P. Ramani & B. E. Young (eds.). Threatened Amphibians of the World. Barcelona, Spain: Lynx Edicions; Gland, Switzerland: IUCN - The World Conservation Union; Arlington, Virginia: Conservation International.

Sukumaran, J., and Holder, M. T. 2010. DendroPy: a Python library for phylogenetic computing. Bioinformatics 26:1569–1571.

Tamura, K., J. Dudley, M. Nei, and S. Kumar. 2007. MEGA4: Molecular Evolutionary Genetic Analysis (MEGA) software version 4.0. Mol. Biol. Evol. 24:1596–1599.

Taylor, E. H. 1962. The Amphibian Fauna of Thailand. Univ. Kansas Sci. Bull. 43:267–99.

Thompson, J. D., T. J. Gibson, F. Plewniak, F. Jeanmougin, and D. G. Higgins. 1997. The CLUSTAL X windows interface: flexible strategies for multiple sequence alignment aided by quality analysis tools. Nucleic Acids Res. 25:4876–4882.

van Bocxlaer, I., K. Roelants, S. D. Biju, J. Nagaraju, and F. Bossuyt. 2006. Late Cretaceous vicariance in Gondwanan amphibians. PLoS ONE 1:e74.

van der Meijden, A., M. Vences, S. Hoegg, R. Boistel, A. Channing, and A. Meyer. 2007. Nuclear gene phylogeny of narrow-mouthed toads (family: Microhylidae) and a discussion of competing hypotheses concerning their biogeographical origins. Mol. Phylogenet. Evol. 44:1017–1030.

Wiens J. J. 2006. Missing data and the design of phylogenetic analyses. J. Biomed. Informatics 39:34–42.

Wiens, J. J., T. N. Engstrom, and P. T. Chippindale. 2006. Rapid diversification, incomplete isolation, and the "speciation clock" in North American salamanders (genus *Plethodon*): testing the hybrid swarm hypothesis of rapid radiation. Evolution 60:2585–2603.

Wiens J. J., and D. S. Moen. 2008. Missing data and the accuracy of Bayesian phylogenetics. J. Syst. Evol. 46:307–314.

Wiley, E. O. 1978. The evolutionary species concept reconsidered. Syst. Zool. 21:17–26.

Winker, K., M. J. Braun, and G. R. Graves. 1996. Voucher specimens and quality control in avian molecular studies. Ibis 138:345–346.

Yates, T. L. 1985. The role of voucher specimens in mammal collections: characterization and funding responsibilities. Acta Zool. Fennica 170:81–82.

Zhao, E.-M., and K. Adler. 1993. Herpetology of China. Society for the Study of Amphibians and Reptiles. Oxford, OH, 522 pp.

Supplementary Figure 1. Maximum-likelihood phylogram estimated from mitochondrial DNA sequences (12S and 16S ribosomal RNA genes) depicting the phylogenetic relationships of other microhylid taxa in relation to *Kaloula*. Note that *K. taprobanica* does not form a clade with other species of *Kaloula.* The outgroup *Callulina* is not shown.

Supplementary Figure 2. Maximum-likelihood phylogram estimated from mitochondrial DNA sequences (12S and 16S ribosomal RNA genes) depicting the phylogenetic relationships of *Kaloula* (Anura: Microhylidae; figure complements Fig. 1). Species groups discussed in the text are highlighted in gray boxes.

Supplementary Figure 3. Uncertainty in disparification through time in *Kaloula*. Panels show disparity-through-time (DTT) plots for $PC1_{phylo}$, $PC2_{phylo}$, $PC3_{phylo}$, and all three axes together ($PC1–3_{phylo}$). Solid black line on DTT plots representent observed disparity based on MCCT; gray polygons represent 95% confidence interval for observed disparity based on post-burnin trees from BEAST analysis.

Supplementary Table 1.—Summary of specimens corresponding to genetic samples included in the study. ABTC = South Australian Museum genetic resources collection; BS-FS = Biotic Survey (David Bickford) field series, deposited at the University of Papuan New Guinea Reference Collection; ACD = Arvin Diesmos field series, specimen deposited at the National Museum of the Philippines; CAS = California Academy of Sciences Herpetological Collections; DCC = David Cannatella field series; DLSUD = deposited in De La Salle (Cavite, Philippines) University Reference collection; DWNP = deposited in University of Malaya Reference Collection; Franky Bossyut personal collection; FMNH = Field Museum of Natural History Herpetological Collections; FRIM = Forest Research Institute of Malaysia Reference Collection; GVAG = Genevieve V. A. Gee field series, deposited at the National Museum of the Philippines; ID = Indraniel Das field number, specimen deposited at Raffles Museum; JS = Jeet Sukumaran field number, specimen deposited at FRIM; KU = University of Kansas Natural History Museum; LSUHC = La Sierra University Herpetological Collections; LSUMZ = Louisiana State University Herpetological Collection; MF = Mike Forstner (Texas State University) frozen tissue collection; MG = Maren Gaulke field series (voucher specimens lost); MM = Madhava Meegaskumbura field series; MZB = Museum Zoologicum Bogoriense, Java, Indonesia; NMNS = National Museum of Natural Science, Taiwan; PNM/CMNH-H = uncatalogued material, deposited in the Cincinnati Museum of Natural History; RMB = Rafe Brown field number, uncataloged specimen deposited at the National Museum of the Philippines; ROM = Royal Ontario Museum; SP = Sabah Parks Collection, Sabah, Borneo, Malaysia; TNHC = Texas Natural History Collections, University of Texas at Austin; TZ = Thomas Ziegler, personal collection; USNM FS = United States National Museum Field Series; ZUEC = Museu de História Natural, Universidade Estadual de Campinas, Brazil.

Species	Voucher	Locality	GenBank Accession Numbers
Chiasmocleis carvalhoi	DCC 3433, Deposited in ZUEC	Brasil, Rio de Janeiro, Seropédica, near Univ. Federal Rural do Rio de Janeiro	JN604510
Austrochaperina sp.	BS-FS 11377	Papua New Guinea	KC822485
Calluella guttata	FMNH 252956	Vietnam, Gia-Lai Province, Ankhe District	KC822482
Calluella guttata	FMNH 252957	Vietnam, Gia-Lai Province, Ankhe District	KC822483
Calluella yunnanensis	FMNH 232988	China, Sichuan, Xichang Region	KC822481
Callulina kreffti	TNHC 62491	Tanzania (pet store)	AY326068
Chaperina fusca	KU 329495	Philippines, Palawan Island, Palawan Province, Municipality of Brooke's Point	KC822495
Cophixalus cheesmanae	BS-FS 11393	Papua New Guinea	EU394373
Cophixalus sp.	TNHC 51333	Papua New Guinea, Morobe	KC822480
Elachistocleis cesari	DCC 3301, Deposited in ZUEC	Brazil, Minas Gerais, Serra do Cipó, 24.2km N of Rio Cipó	JN604511
Gastrophryne olivacea	TNHC 61952	USA, Texas, San Saba, Colorado Bend State Park	AY326066
Glyphoglossus molossus	CAS 221608	Myanmar, Mandalay Division, Thazi Township, Yin Mar Bin Village	KC822484
Kalophrynus pleurostigma	KU 326458	Philippines, Mindanao Island	KC822497
Kalophrynus pleurostigma	KU 326459	Philippines, Bohol Island, Bohol Province, Municipality of Antequera	KC822496
Kaloula baleata Java & Bali	JAM 3232	Indonesia, Bali Island, Propinsi Bali, Renon (a suburb of Denpasar)	KC822570
Kaloula conjuncta conjuncta	ACD 769	Philippines, Luzon Island, Palola	KC822537
Kaloula conjuncta conjuncta	ACD 996	Philippines, Mindanao Island, Bukidnon Province, Malagaylay	KC822536
Kaloula conjuncta conjuncta	KU 301849	Philippines, Semirara Island, Antique Province, Municipality of Caluya	KC822532
Kaloula conjuncta conjuncta	KU 301854	Philippines, Semirara Island, Antique Province, Municipality of Caluya	KC822533
Kaloula conjuncta conjuncta	KU 303279	Philippines, Polillo Island, Quezon Province, Municipality of Polillo	KC822527

Kaloula conjuncta conjuncta	KU 320031	Philippines, Luzon Island, Laguna Province, Municipality of Los Banos, Mt. Makiling	KC822538
Kaloula conjuncta conjuncta	KU 323280	Philippines, Mindoro Island, Mindoro Occidental Province, Municipality of Sablayan	KC822535
Kaloula conjuncta conjuncta	RMB 509	Philippines, Mindoro Island, Mindoro Oriental Province, Municipality of Calapan	KC822534
Kaloula conjuncta conjuncta	TNHC 59628	Philippines, Luzon Island, Quezon Province, Municipality of Tayabas	KC822539
Kaloula conjuncta conjuncta	TNHC 62972	Philippines, Luzon Island, Albay Province, Municipality of Malinao, Mt. Malinao	KC822528
Kaloula conjuncta conjuncta	TNHC 62973	Philippines, Luzon Island, Albay Province, Municipality of Malinao, Mt. Malinao	KC822526
Kaloula conjuncta conjuncta	TNHC 62975	Philippines, Luzon Island, Albay Province, Muncipality of Tabaco City	KC822530
Kaloula conjuncta conjuncta	TNHC 62976	Philippines, Luzon Island, Albay Province, Muncipality of Tabaco City	KC822529
Kaloula conjuncta conjuncta	TNHC 62986	Philippines, Luzon Island, Sorsogon Province, Municipality of Irosin	KC822531
Kaloula conjuncta meridionalis	KU 309658	Philippines, Camiguin Sur Island, Camiguin Sur Province, Municipality of Mambajao	KC822588
Kaloula conjuncta meridionalis	TNHC 59632	Philippines, Mindanao Island, Davao del Sur Province, Municipality of Toril	KC822521
Kaloula conjuncta meridionalis	TNHC 59635	Philippines, Mindanao Island, Davao del Sur Province, Municipality of Toril	KC822520
Kaloula conjuncta meridionalis	TNHC 59636	Philippines, Mindanao Island, Davao del Sur Province, Municipality of Toril	KC822522
Kaloula conjuncta meridionalis	TNHC 59669	Philippines, Mindanao Island, Davao del Sur Province, Municipality of Toril	KC822524
Kaloula conjuncta meridionalis	TNHC 59670	Philippines, Mindanao Island, Davao del Sur Province, Municipality of Toril	KC822523
Kaloula conjuncta meridionalis	TNHC 59870	Philippines, Mindanao Island, Davao City Province, Municipality of Calinan	KC822525
Kaloula conjuncta negrosensis	KU 328639	Philippines, Negros Island, Negros Oriental Province, Municipality of Dumaguete City	KC822518
Kaloula conjuncta negrosensis	KU 328640	Philippines, Negros Island, Negros Oriental Province, Municipality of Dumaguete City	KC822519
Kaloula kalingensis	KU 328643	Philippines, Palaui Island, Cagayan Province	KC822603
Kaloula kalingensis	KU 328644	Philippines, Palaui Island, Cagayan Province	KC822604
Kaloula kalingensis	RMB 2210	Philippines, Luzon Island, Kalinga Province, Municipality of Balbalan, Mt. Magdallao	KC822609
Kaloula kalingensis	RMB 3137	Philippines, Luzon Island, Kalinga Province, Municipality of Balbalan	KC822610
Kaloula kalingensis	TNHC 59647	Philippines, Luzon Island, Kalinga Province, Municipality of Balbalan	KC822608
Kaloula kalingensis	TNHC 60118	Philippines, Luzon Island, Kalinga Province, Municipality of Balbalan	KC822605
Kaloula kokacii	KU 313824	Philippines, Luzon Island, Camarines del Norte Province, Municipality of Labo, Mt. Labo	KC822596
Kaloula kokacii	KU 328629	Philippines, Luzon Island, Camarines Sur Province, Municipality of Naga, Mt. Isarog	KC822595
Kaloula kokacii	KU 328630	Philippines, Luzon Island, Albay Province, Mt. Malinao	KC822590
Kaloula kokacii	KU 328634	Philippines, Luzon Island, Quezon Province, Municipality of Atimonan	KC822593
Kaloula kokacii	TNHC 62684	Philippines, Luzon Island, Camarines Sur Province, Muncipality of Naga City, Mt. Isarog	KC822594
Kaloula kokacii	TNHC 62685	Philippines, Luzon Island, Albay Province, Municipality of Tiwi, Mt. Malinao	KC822589
Kaloula kokacii	TNHC 62687	Philippines, Luzon Island, Albay Province, Municipality of Malinao, Mt. Malinao	KC822591

Kaloula kokacii	TNHC 62688	Philippines, Luzon Island, Sorsogon Province, Municipality of Irosin	KC822592
Kaloula mediolineata	FMNH 265819	Thailand, Ubon Ratchatani, Nam Yuen	KC822510
Kaloula mediolineata	KU 328280	Thailand, Sakaerat, Nakhorn Ratchasima Province, Wang Nam Kieow district	KC822509
Kaloula mediolineata	KU 328285	Thailand, Sakaerat, Nakhorn Ratchasima Province, Wang Nam Kieow district	KC822508
Kaloula mediolineata	ROM 32838	Vietnam, Gia Lai, Krong Pa	KC822571
Kaloula picta	ACD 1206	Philippines, Palawan Island	KC822541
Kaloula picta	ACD 1242	Philippines, Leyte Island, Leyte Province	KC822563
Kaloula picta	ACD 1261	Philippines, Palawan Island, Palawan Province, Municipality of Puerto Princesa City	KC822542
Kaloula picta	ACD 1304	Philippines, Palawan Island	KC822540
Kaloula picta	ACD 1390	Philippines, Luzon Island, Pampanga Province, Municipality of Angeles City	KC822546
Kaloula picta	ACD 1501	Philippines, Leyte Island, Leyte Province, Muncipality of Baybay	KC822562
Kaloula picta	DLSUD 020	Philippines, Luzon Island, Cavite Province, Mt. Palay-Palay	KC822552
Kaloula picta	DLSUD 021	Philippines, Luzon Island, Cavite Province, Mt. Palay-Palay	KC822553
Kaloula picta	KU 301873	Philippines, Camiguin Sur Island, Camiguin Sur Province, Municipality of Mambajao	KC822565
Kaloula picta	KU 326261	Philippines, Luzon Island, Laguna Province, Municipality of Los Banos	KC822554
Kaloula picta	KU 326262	Philippines, Luzon Island, Laguna Province, Municipality of Los Banos	KC822555
Kaloula picta	KU 326265	Philippines, Luzon Island, Quezon Province, Municipality of Atimonan	KC822548
Kaloula picta	RMB 4223	Philippines, Luzon Island, Cagayan Province, Municipality of Gattaran	KC822551
Kaloula picta	RMB 4224	Philippines, Luzon Island, Cagayan Province, Municipality of Gattaran	KC822550
Kaloula picta	RMB 4294	Philippines, Leyte Island, Leyte Province, Municipality of Tacloban	KC822564
Kaloula picta	RMB 4344	Philippines, Leyte Island, Leyte Province, Muncipality of Baybay	KC822561
Kaloula picta	TNHC 56430	Philippines, Bohol Island, Bohol Province, Municipality of Bilar	KC822559
Kaloula picta	TNHC 56431	Philippines, Bohol Island, Bohol Province, Municipality of Bilar	KC822560
Kaloula picta	TNHC 56468	Philippines, Cebu Island, Cebu Province, Municipality of Cebu City	KC822543
Kaloula picta	TNHC 59653	Philippines, Mindanao Island, Davao del Sur Province, Municipality of Toril	KC822558
Kaloula picta	TNHC 59654	Philippines, Mindanao Island, Davao del Sur Province, Municipality of Toril	KC822557
Kaloula picta	TNHC 59871	Philippines, Mindanao Island, Davao del Sur Province, Municipality of Toril	KC822556
Kaloula picta	TNHC 62470	Philippines, Luzon Island, Albay Province, Muncipality of Tabaco City	KC822549
Kaloula picta	TNHC 62471	Philippines, Luzon Island, Zambales Province, Municipality of Olongapo	KC822547
Kaloula picta	USNM FS-56932	Philippines, Polillo Island, Quezon Province, Municipality of Polillo	KC822545
Kaloula picta	USNM FS-56936	Philippines, Polillo Island, Quezon Province, Municipality of Polillo	KC822544
Kaloula pulchra	ACD 1538	Thailand	KC822621
Kaloula pulchra	FMNH 255128	Lao PDR, Khammouan Province, Boualapha District	KC822620
Kaloula pulchra	FMNH 255129	Lao PDR, Bolikhamxay Province, Thaphabat District	KC822619
Kaloula pulchra	JAM 1753	Malaysia, Selangor Province, Ulu Gombak Field Studies Centre	AY326064
Kaloula pulchra	JAM 1857	Malaysia, Pahang Province, Tioman Island	KC822615
Kaloula pulchra	LSUHC 3869	West Malaysia, Pahang, Pulau Tioman, Tekek-Juara Trail	KC822575
Kaloula pulchra	LSUHC 3870	West Malaysia	KC852906

Kaloula pulchra	MF 0766	Indonesia, Sumatra Island	KC822623
Kaloula pulchra	MF 0812	Indonesia, Sumatra Island	KC822624
Kaloula pulchra	NMNS 3208	China	KC822614
Kaloula pulchra	TNHC 59422	Indonesia, Sulawesi Island, Propinsi Sulawesi Selatan, SE quadrant of Ujung Pandang, Kelurahan Karung-karung	KC822618
Kaloula pulchra	TNHC 59423	Indonesia, Sulawesi Island, Propinsi Sulawesi Selatan, SE quadrant of Ujung Pandang, Kelurahan Karung-karung	KC822617
Kaloula pulchra	TZ 629	Vietnam	KC822622
Kaloula rigida	ACD 1570	Philippines, Luzon Island, Benguet Province, Baguio City	KC822636
Kaloula rigida	ACD 1954	Philippines, Luzon Island, Isabela Province, Municipality of San Mariano	KC822631
Kaloula rigida	ACD 2043	Philippines, Luzon Island, Isabela Province, Municipality of San Mariano	KC822632
Kaloula rigida	ACD 2044	Philippines, Luzon Island, Isabela Province, Municipality of San Mariano	KC822634
Kaloula rigida	ACD 2032, Deposited in PNM	Philippines, Luzon Island, Kalinga Province	KC822633
Kaloula rigida	ACD 660	Philippines, Luzon Island, Sierra Madres Mountain Range	KC822626
Kaloula rigida	ACD 756	Philippines, Luzon Island, Kalinga Province, Municipality of Balbalan	KC822628
Kaloula rigida	KU 326470	Philippines, Luzon Island, Isabela Province, Municipality of San Mariano	KC822635
Kaloula rigida	KU 328628	Philippines, Luzon Island, Cagayan Province, Municipality of Pagudpud	KC822627
Kaloula rigida	RMB 4226	Philippines, Luzon Island, Cagayan Province, Municipality of Gattaran	KC822625
Kaloula rigida	TNHC 59644	Philippines, Luzon Island, Kalinga Province, Municipality of Balbalan	KC822629
Kaloula rigida	TNHC 60119	Philippines, Luzon Island, Kalinga Province, Municipality of Balbalan	KC822630
Kaloula sp. nov. East Luzon	ACD 1692	Philippines, Luzon Island, Laguna Province, Mt. Makiling	KC822602
Kaloula sp. nov. East Luzon	ACD 2479	Philippines, Luzon Island, Sierra Madres Mountain Range	KC822606
Kaloula sp. nov. East Luzon	ACD 650	Philippines, Luzon Island, Isabela Province, Municipality of Palanan	KC822607
Kaloula sp. nov. East Luzon	ACD 943	Philippines, Luzon Island, Laguna Province, Municipality of Los Banos, Mt. Makiling	KC822597
Kaloula sp. nov. East Luzon	ACD 945	Philippines, Luzon Island, Laguna Province, Municipality of Los Banos, Mt. Makiling	KC822598
Kaloula sp. nov. East Luzon	FMNH 267555	Philippines, Luzon Island, Laguna Province, Municipality of Los Banos, Mt. Makiling	KC822599
Kaloula sp. nov. East Luzon	RMB 750, Deposited in CMNH	Philippines, Luzon Island, Aurora Province, Aurora Memorial National Park	KC822601
Kaloula sp. nov. East Luzon	RMB 783, Deposited in CMNH	Philippines, Luzon Island, Aurora Province, Aurora Memorial National Park	KC822600
Kaloula sp. nov. Palawan	ACD 1303	Philippines, Palawan Island	KC822582
Kaloula sp. nov. Palawan	ACD 1307	Philippines, Palawan Island	KC822583
Kaloula sp. nov. Panay	MG 0000	Philippines, Panay Island, Antique Province, Municipality of Sibalom	KC822611
Kaloula sp. nov. Panay	MG 0001	Philippines, Panay Island, Antique Province, Municipality of Sibalom	KC822612
Kaloula sp. nov. Panay	MG 0002	Philippines, Panay Island, Antique Province, Municipality of Sibalom	KC822613
Kaloula sp. nov. Peninsular Malaysia	DWNP 975	Malaysia	KC822580
Kaloula sp. nov. Peninsular Malaysia	FRIM 1066	Malaysia	KC822579
Kaloula sp. nov. Peninsular Malaysia	ID 8317	Borneo	KC822581

Kaloula sp. nov. Peninsular Malaysia	LSUHC 5074	West Malaysia, Kedah, Gubir	KC822576
Kaloula sp. nov. Peninsular Malaysia	LSUHC 5712	West Malaysia, Pahang, Pualu Aceh	KC822577
Kaloula sp. nov. Peninsular Malaysia	LSUHC 6156	East Malaysia, Sabah, Sepilok Jungle Resort	KC822578
Kaloula sp. nov. Samar & Leyte	KU 310699	Philippines, Samar Island, Eastern Samar Province, Municipality of Taft	KC822587
Kaloula sp. nov. Samar & Leyte	KU 328632	Philippines, Leyte Island, Leyte Province, Muncipality of Danao	KC822584
Kaloula sp. nov. Samar & Leyte	KU 328633	Philippines, Leyte Island, Leyte Province, Muncipality of Danao	KC822585
Kaloula sp. nov. Samar & Leyte	KU 328645	Philippines, Leyte Island, Leyte Province, Muncipality of Baybay	KC822586
Kaloula sp. nov. Sibuyan	GVAG 253	Philippines, Panay Island, Antique Province, Municipality of Sibalom	KC822515
Kaloula sp. nov. Sibuyan	GVAG 255	Philippines, Panay Island, Antique Province, Municipality of Sibalom	KC822514
Kaloula sp. nov. Sibuyan	KU 328607	Philippines, Sibuyan Island, Romblon Province	KC822511
Kaloula sp. nov. Sibuyan	KU 328608	Philippines, Sibuyan Island, Romblon Province	KC822512
Kaloula sp. nov. Sibuyan	MG 0012	Philippines, Panay Island, Antique Province	KC822513
Kaloula sp. nov. Sibuyan	TNHC 56341	Philippines, Panay Island, Antique Province, Mt. Baloy	KC822516
Kaloula sp. nov. Sibuyan	TNHC 56343	Philippines, Panay Island, Antique Province, Mt. Baloy	KC822517
Kaloula sp. nov. Sulawesi	JAM 3853	Indonesia, Propinsi Sulawesi Tengah, Kabupaten Poso, Kecamatan Una Una, Pulau Batudaka (Togian Islands), Desa Wakai, Air Terjun Tanimpo	KC822568
Kaloula sp. nov. Sulawesi	LSUMZ 83998	Indonesia, Sulawesi Island, Propinsi Sulawesi Tengah, Kabupaten Donggala, Kecamatan Parigi, Desa Binanggi	KC822566
Kaloula sp. nov. Sulawesi	LSUMZ 83999	Indonesia, Sulawesi Island, Propinsi Sulawesi Utara, Bogani Nani Wartabone National Park, Kabupaten Bolaang Mongondow, Desa Torout	KC822567
Kaloula sp. nov. Vietnam	ROM 32925	Vietnam, Gia Lai, Krong Pa	KC822572
Kaloula sp. nov. Vietnam	ROM 32932	Vietnam, Gia Lai, Krong Pa	KC822573
Kaloula sp. nov. Vietnam	ROM 32943	Vietnam, Gia Lai, Krong Pa	KC822574
Kaloula sp. nov. Vietnam	TNHC 67086	Indonesia, Java Island, Jawa Barat	KC822569
Kaloula taprobanica	FB 102	Sri Lanka	KC822506
Kaloula taprobanica	MM 2871	Sri Lanka	KC822505
Kaloula verrucosa	NMNS 3246	China	KC822507
Kaloula walteri	ACD 994	Philippines, Mindanao Island, Bukidnon Province, Malagaylay	KC822640
Kaloula walteri	ACD 996	Philippines, Mindanao Island, Bukidnon Province, Malagaylay	KC822536
Kaloula walteri	KU 303285	Philippines, Polillo Island, Quezon Province, Municipality of Polillo	KC822642
Kaloula walteri	KU 327338	Philippines, Luzon Island, Quezon Province, Municipality of Lucban	KC822639
Kaloula walteri	RMB 3701	Philippines, Luzon Island, Quezon Province, Municipality of Tayabas	KC822641
Kaloula walteri	TNHC 59667	Philippines, Luzon Island, Quezon Province, Municipality of Lucban	KC822637
Kaloula walteri	TNHC 60116	Philippines, Luzon Island, Quezon Province, Municipality of Lucban	KC822638
Metaphrynella pollicarus	JS 00787	Malaysia, Genting	KC822502
Metaphrynella pollicarus	JS 00794	Malaysia, Genting	KC822503
Metaphrynella sundana	FMNH 231199	Malaysia, Sabah, Lahad Datu District	KC822500
Metaphrynella sundana	FMNH 231203	Malaysia, Sabah, Lahad Datu District	KC822501
Metaphrynella sundana	ID 7350	Malaysia, Borneo, Poring	KC822499

Metaphrynella sundana	RMBR 246, Deposited in ZRC	Malaysia, Borneo	EU394358
Microhyla achatina	RMB 2629, Deposited at MZB	Indonesia, Java Island	KC822492
Microhyla berdmorei	JAM 1995, Deposited in SP	Malaysia, Perlis Province, 9 km W Padang Besar, FRIM field station	EU394354
Microhyla ornata	JAM 1991, Deposited in SP	Malaysia, Perlis Province, 9 km W Padang Besar, FRIM field station	KC822490
Micryletta inornata	FMNH 255121	Lao PDR, Khammouan Province, Boualapha District	KC822494
Micryletta inornata	FMNH 255123	Lao PDR, Bolikhamxay Province, Thaphabat District	KC822493
Nelsonophryne aequatorialis	KU 202919	Ecuador, Loja	AY326067
Oreophryne annulata	PNM/CMNH-H 1366	Philippines, Mindanao Island, Davao del Sur Province, Mt. Apo	KC822488
Oreophryne brachypus	ABTC 50081	Papua New Guinea, New Britain Province, New Britain Island	KC822487
Oreophryne variabilis	TNHC 58922	Indonesia, Sulawesi Island, Propinsi Sulawesi Selatan	KC822489
Ramanella obscura	MM 5980	Sri Lanka	KC822504
Scaphiophryne marmorata	TNHC84936	Madagascar	EU394357
Scaphiophryne madagascariensis	TNHC 64007	Madagascar (pet trade)	KC822475

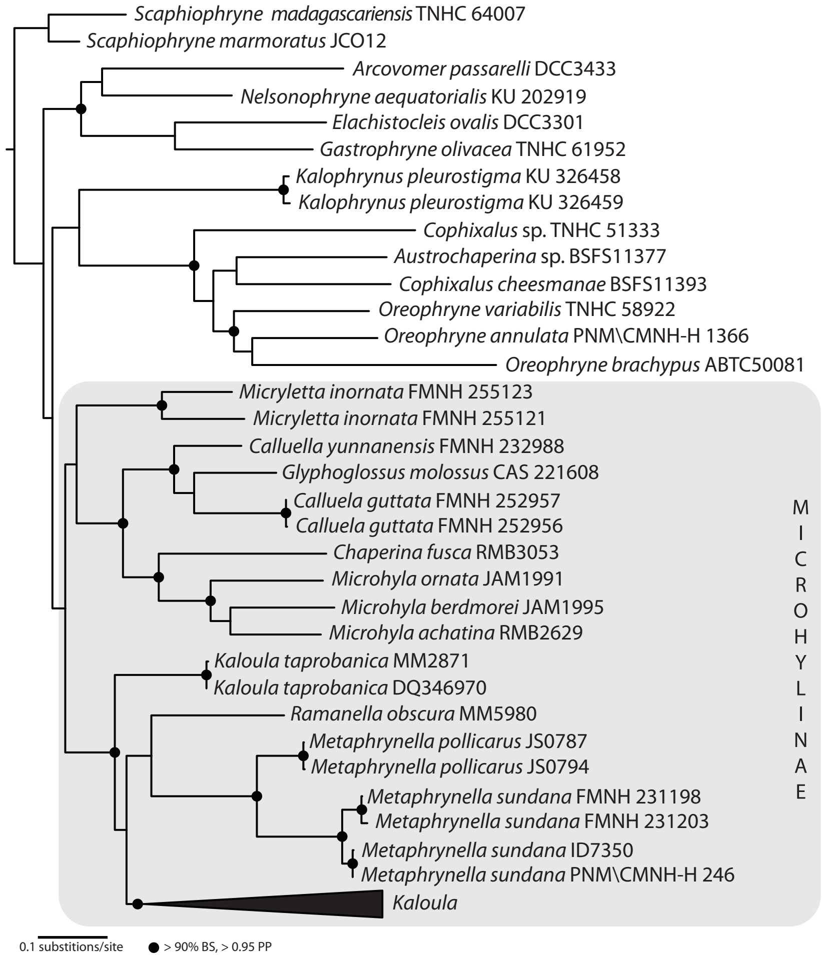
Scaphiophryne madagascariensis TNHC 64007
Scaphiophryne marmoratus JCO12
Arcovomer passarelli DCC3433
Nelsonophryne aequatorialis KU 202919
Elachistocleis ovalis DCC3301
Gastrophryne olivacea TNHC 61952
Kalophrynus pleurostigma KU 326458
Kalophrynus pleurostigma KU 326459
Cophixalus sp. TNHC 51333
Austrochaperina sp. BSFS11377
Cophixalus cheesmanae BSFS11393
Oreophryne variabilis TNHC 58922
Oreophryne annulata PNM\CMNH-H 1366
Oreophryne brachypus ABTC50081
Micryletta inornata FMNH 255123
Micryletta inornata FMNH 255121
Calluella yunnanensis FMNH 232988
Glyphoglossus molossus CAS 221608
Calluela guttata FMNH 252957
Calluela guttata FMNH 252956
Chaperina fusca RMB3053
Microhyla ornata JAM1991
Microhyla berdmorei JAM1995
Microhyla achatina RMB2629
Kaloula taprobanica MM2871
Kaloula taprobanica DQ346970
Ramanella obscura MM5980
Metaphrynella pollicarus JS0787
Metaphrynella pollicarus JS0794
Metaphrynella sundana FMNH 231198
Metaphrynella sundana FMNH 231203
Metaphrynella sundana ID7350
Metaphrynella sundana PNM\CMNH-H 246
Kaloula
MICROHYLINAE
0.1 substitions/site
> 90% BS, > 0.95 PP

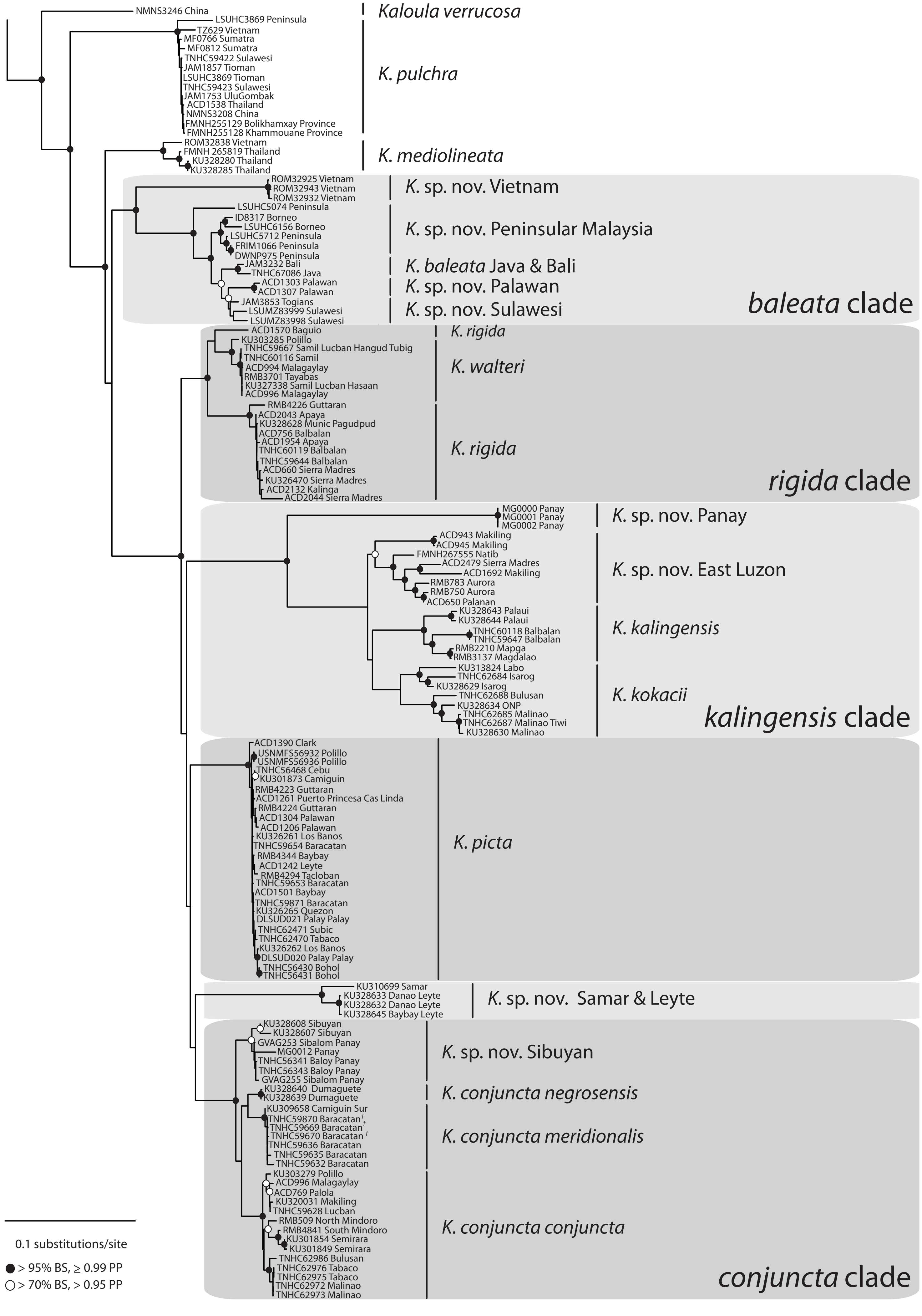

NMNS3246 China
Kaloula verrucosa
LSUHC3869 Peninsula
TZ629 Vietnam
MF0766 Sumatra
MF0812 Sumatra
TNHC59422 Sulawesi
JAM1857 Tioman
LSUHC3869 Tioman
TNHC59423 Sulawesi
JAM1753 UluGombak
ACD1538 Thailand
NMNS3208 China
FMNH255129 Bolikhamxay Province
FMNH255128 Khammouane Province
K. pulchra
ROM32838 Vietnam
FMNH 265819 Thailand
KU328280 Thailand
KU328285 Thailand
K. mediolineata
ROM32925 Vietnam
ROM32943 Vietnam
ROM32932 Vietnam
K. sp. nov. Vietnam
LSUHC5074 Peninsula
ID8317 Borneo
LSUHC6156 Borneo
LSUHC5712 Peninsula
FRIM1066 Peninsula
DWNP975 Peninsula
K. sp. nov. Peninsular Malaysia
JAM3232 Bali
TNHC67086 Java
K. baleata Java & Bali
ACD1303 Palawan
ACD1307 Palawan
K. sp. nov. Palawan
JAM3853 Togians
LSUMZ83999 Sulawesi
LSUMZ83998 Sulawesi
K. sp. nov. Sulawesi
baleata clade
ACD1570 Baguio
K. rigida
KU303285 Polillo
TNHC59667 Samil Lucban Hangud Tubig
TNHC60116 Samil
ACD994 Malagaylay
RMB3701 Tayabas
KU327338 Samil Lucban Hasaan
ACD996 Malagaylay
K. walteri
RMB4226 Guttaran
ACD2043 Apaya
KU328628 Munic Pagudpud
ACD756 Balbalan
ACD1954 Apaya
TNHC60119 Balbalan
TNHC59644 Balbalan
ACD660 Sierra Madres
KU326470 Sierra Madres
ACD2132 Kalinga
ACD2044 Sierra Madres
K. rigida
rigida clade
MG0000 Panay
MG0001 Panay
MG0002 Panay
K. sp. nov. Panay
ACD943 Makiling
ACD945 Makiling
FMNH267555 Natib
ACD2479 Sierra Madres
ACD1692 Makiling
RMB783 Aurora
RMB750 Aurora
ACD650 Palanan
K. sp. nov. East Luzon
KU328643 Palaui
KU328644 Palaui
TNHC60118 Balbalan
TNHC59647 Balbalan
RMB2210 Mapga
RMB3137 Magdalao
K. kalingensis
KU313824 Labo
TNHC62684 Isarog
KU328629 Isarog
TNHC62688 Bulusan
KU328634 ONP
TNHC62685 Malinao
TNHC62687 Malinao Tiwi
KU328630 Malinao
K. kokacii
kalingensis clade
ACD1390 Clark
USNMFS56932 Polillo
USNMFS56936 Polillo
TNHC56468 Cebu
KU301873 Camiguin
RMB4223 Guttaran
ACD1261 Puerto Princesa Cas Linda
RMB4224 Guttaran
ACD1304 Palawan
ACD1206 Palawan
KU326261 Los Banos
TNHC59654 Baracatan
RMB4344 Baybay
ACD1242 Leyte
RMB4294 Tacloban
TNHC59653 Baracatan
ACD1501 Baybay
TNHC59871 Baracatan
KU326265 Quezon
DLSUD021 Palay Palay
TNHC62471 Subic
TNHC62470 Tabaco
KU326262 Los Banos
DLSUD020 Palay Palay
TNHC56430 Bohol
TNHC56431 Bohol
K. picta
KU310699 Samar
KU328633 Danao Leyte
KU328632 Danao Leyte
KU328645 Baybay Leyte
K. sp. nov. Samar & Leyte
KU328608 Sibuyan
KU328607 Sibuyan
GVAG253 Sibalom Panay
MG0012 Panay
TNHC56341 Baloy Panay
TNHC56343 Baloy Panay
GVAG255 Sibalom Panay
K. sp. nov. Sibuyan
KU328640 Dumaguete
KU328639 Dumaguete
K. conjuncta negrosensis
KU309658 Camiguin Sur
TNHC59870 Baracatan†
TNHC59669 Baracatan†
TNHC59670 Baracatan†
TNHC59636 Baracatan
TNHC59635 Baracatan
TNHC59632 Baracatan
K. conjuncta meridionalis
KU303279 Polillo
ACD996 Malagaylay
ACD769 Palola
KU320031 Makiling
TNHC59628 Lucban
RMB509 North Mindoro
RMB4841 South Mindoro
KU301854 Semirara
KU301849 Semirara
TNHC62986 Bulusan
TNHC62976 Tabaco
TNHC62975 Tabaco
TNHC62972 Malinao
TNHC62973 Malinao
K. conjuncta conjuncta
conjuncta clade
0.1 substitutions/site
● > 95% BS, ≥ 0.99 PP
○ > 70% BS, > 0.95 PP

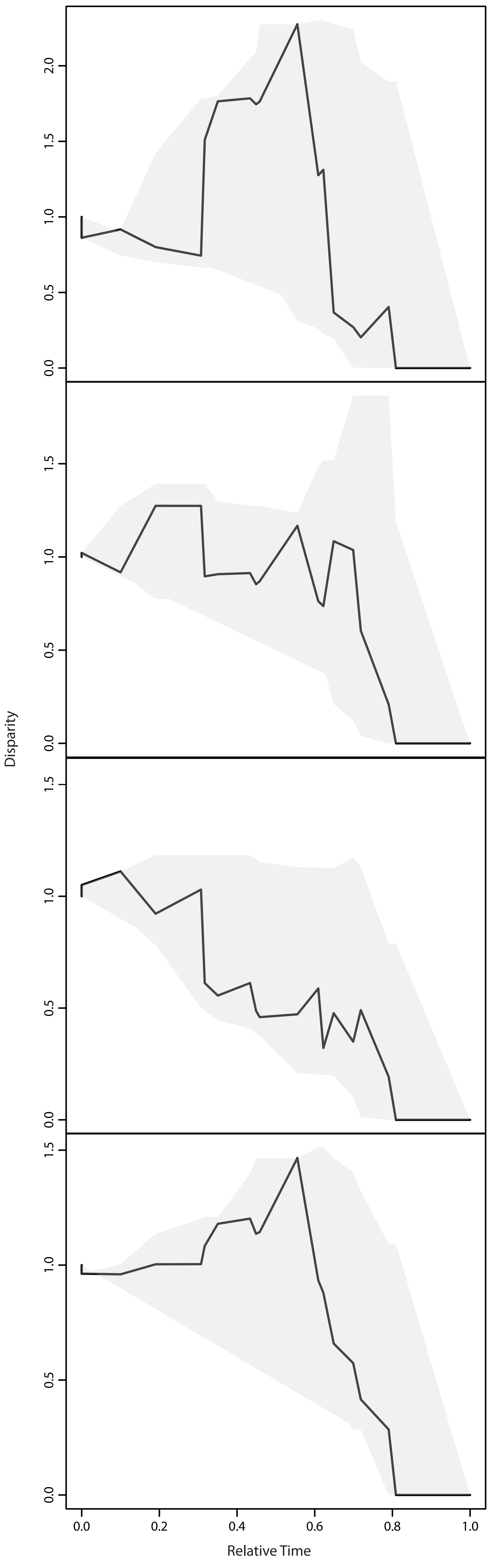

Disparity
Relative Time
0.0
0.5
1.0
1.5
2.0
0.0
0.2
0.4
0.6
0.8
1.0
